# Age-dependent axonal dysfunctions and altered sharp-wave ripple oscillations in *Scn1a*^*+/−*^ mice

**DOI:** 10.1016/j.isci.2026.116784

**Published:** 2026-07-21

**Authors:** Raquel Lascorz, Fabian C. Roth

**Affiliations:** 1 Division of Physiology, Department of Molecular Medicine, Institute of Basic Medical Sciences, University of Oslo, Oslo, Norway

**Keywords:** Dravet syndrome, sharp wave ripple, SCN1A, NaV1.1, axonal recording, parvalbumin-expressing interneurons, mice

## Abstract

Mice with *Scn1a* haploinsufficiency replicate symptoms of Dravet syndrome (DS), a rare childhood disease characterized by febrile seizures at an early age. As adults, patients with DS suffer from epilepsy, motor dysfunction, psychological disorders, and cognitive disabilities. Patients with DS carry loss-of-function variants in the *SCN1A* gene, which encodes the voltage-gated sodium channel subunit Na_V_1.1. As shown in mice, this subunit is mainly expressed in GABAergic cells, especially parvalbumin-expressing interneurons (PV-INs). Consistent with this, interneuron-driven network activities, such as sharp wave ripple oscillations (SPW-R), are altered in *Scn1a*-deficient mice. By studying network and PV-IN activity during SPW-R in hippocampal slices from *Scn1a*^*+/−*^ mice, we identified age-dependent impairments in ripple oscillation frequencies and in rhythmic action potential (AP) firing in PV-INs, accompanied by alterations in somatic and axonal AP waveforms. This age-dependent increase in hippocampal cellular and network abnormalities may contribute to the persisting cognitive deficits associated with *Scn1a* haploinsufficiency.

## Highlights

• *Scn1a*^*+/−*^ mice show an age-dependent reduction in ripple frequencies *in vitro*
• Rhythmic PV-IN firing and synaptic inhibition are altered only in young adults
• Action potential deficits in PV-INs accompany changes in ripple oscillations
• Proximal PV-IN axons of young adult *Scn1a*^*+/−*^ mice show the most severe AP deficits

## Graphical abstract

**Figure:**
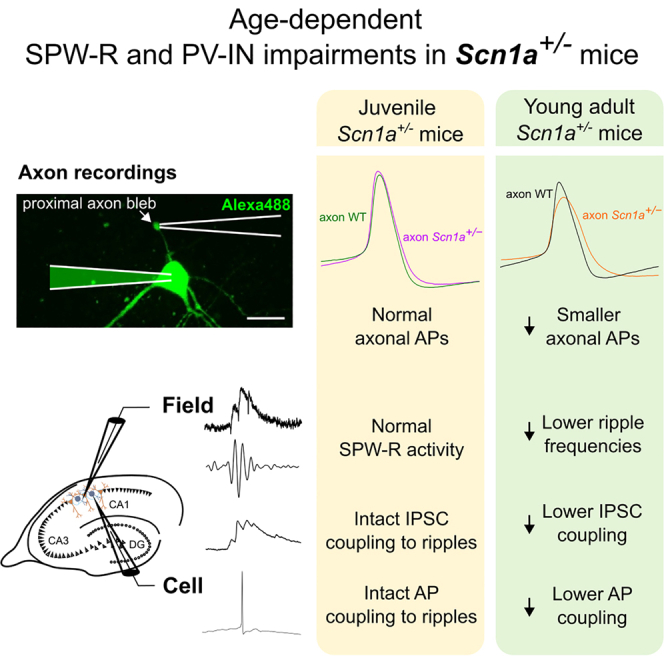

## Teaser

Neuroscience; Cellular neuroscience

## Introduction

Dravet syndrome (DS) is a rare epileptic encephalopathy and a public health problem with an estimated prevalence between ∼1:15000 and 1:41000 for children in the USA.^1^ In 1978, it was described as severe myoclonic epilepsy of infancy (SMEI)^2^ and later named Dravet syndrome after the author in 1989. The onset of the disease occurs at ∼5–8 months of age and is typically characterized by fever-induced clonic, generalized seizures with longer durations than simple febrile seizures.^3^^,^^4^ Dravet-associated epilepsy progresses from febrile to spontaneously occurring seizures, and patients with DS show a high rate of mortality (∼15%) caused by severe status epilepticus, accidents, infections, and sudden unexpected death in epilepsy (SUDEP). As adults, patients with DS often suffer from psychological disorders as well as motor and cognitive dysfunctions.^4^^,^^5^

DS is associated with loss-of-function mutations in the *SCN1A* gene, which encodes the type 1 neuronal voltage-gated sodium (Na_V_) channel α subunit Na_V_1.1. This subunit is predominantly expressed in axons of GABAergic interneurons.^6^ With the generation of genetically modified mice, Yu et al.^7^ demonstrated that a full deletion or haploinsufficiency of *Scn1a* replicates core Dravet symptoms, linking interneuron-specific reductions in sodium currents and fast-spiking activity to the occurrence of spontaneous seizures. Numerous following studies confirmed that the deficits in the action potential (AP) generation in fast-spiking GABAergic interneurons are consistent with reduced Na_V_ channel activity.^4^^,^^8^^,^^9^^,^^10^ These fast-spiking interneurons typically represent parvalbumin (PV)-expressing interneurons (PV-INs) that were shown to strongly express *Scn1a* as opposed to the levels found in somatostatin (SOM)-expressing interneurons and other PV/SOM-negative cells.^11^

Consistent with these findings, it was hypothesized that a *Scn1a* insufficiency may cause dysfunctions in network activity that involve high PV-IN activity. Indeed, DS-mice were found to have impaired sharp wave ripple oscillations (SPW-R),^12^ which are associated with memory consolidation during resting states.^13^^,^^14^^,^^15^^,^^16^ It was shown that spontaneously generated SPW-R represent field activity of a subset of simultaneously active neurons, which is dominated by strong phasic inhibition provided by PV-INs.^17^^,^^18^^,^^19^^,^^20^^,^^21^ These specialized interneurons provide cortical networks with feedforward and feedback inhibition and organize the temporal structure of the network activity during different behavioral states.^22^ During SPW-R in the hippocampus, synchronized fast firing of PV-INs leads to strong, rhythmic perisomatic inhibition of surrounding pyramidal cells, which creates repetitive “windows of opportunity” for AP generation, thereby entraining pyramidal cells into phase-locked firing during ripple cycles.^23^ Accordingly, the high-frequency (150–300 Hz) fluctuations in the field potentials (the ripple oscillations) are believed to be directly generated by the compound inhibitory postsynaptic currents (IPSCs). Altered generation or waveforms of APs in PV-INs are thus expected to change the timing and synchrony of compound IPSCs and the generation of ripple oscillations in the field potentials, which will also affect the temporal organization of the network during PV-IN-dependent activities.^20^^,^^24^^,^^25^

In their comprehensive study, Kaneko et al.^24^ analyzed the age-dependent expression of AP deficits in PV-INs associated with *Scn1a* haploinsufficiency in the somatosensory cortex of *Scn1a*^+/−^ mice. They confirmed earlier findings^8^ stating that the fast-spiking phenotype was most strongly affected at juvenile age (P16-21) before mortality (SUDEP) onset and recovered to normal spiking at young adult age (P35-56) despite a persistence of altered inhibitory synaptic transmission compared to wild-type (WT) mice. They also found that juvenile *Scn1a*^+/−^ mice showing stronger dysfunctions were more prone to die prematurely.

Here, we studied the missing link between *in vivo* data on SPW-R and known cellular dysfunctions studied *in vitro* to better understand the underpinnings of altered network activity that is affecting cognitive performance. We investigated whether there is a similar age-dependent expression of deficits in PV-INs and related network activity in the hippocampus of *Scn1a*^+/−^ mice during spontaneous SPW-R. For this, we made use of a hippocampal slice preparation to study axonal, cellular, and field activity simultaneously using a combination of advanced electrophysiological recording techniques.^25^^,^^26^^,^^27^^,^^28^ We indeed found age-dependent changes in SPW-R activity for *Scn1a*^+/−^ mice but these impairments became stronger with increasing age rather than recovered. This observation is opposite to the previously described transient expression of fast-spiking deficits in PV-INs.^8^^,^^10^^,^^24^^,^^29^ Consistent with *in vivo* data,^12^ we found that SPW-R in CA1 show a reduced ripple frequency in slices prepared from young adult (P30-37) *Scn1a*^+/−^ mice. Juvenile mice (P19-23), in turn, did not show such differences in SPW-R, suggesting an age-dependent development of pathological hippocampal activity associated with *Scn1a* haploinsufficiency. These changes were accompanied by differences in rhythmic AP firing in PV-INs and in their inhibitory synaptic output to pyramidal cells. In addition, both stimulation-induced and spontaneous APs showed more strongly reduced amplitudes, reduced maximum rising slope, and increased width in young adult *Scn1a*^+/−^ mice, all consistent with reduced Na_V_ channel activity. Direct recordings from proximal PV-IN axons revealed similar changes in AP waveforms in young adult, but not in juvenile *Scn1a*^+/−^ mice. A partial block of the active Na_V_ channel pool by tetrodotoxin (TTX) further indicated a stronger difference in active Na_V_ channels in young adult *Scn1a*^*+/−*^ compared to WT mice. Together, we find age-dependent alterations of neuronal and network activity toward older ages in this mouse model of DS, which appear independently of the previously described fast-spiking deficit.^30^

## Results

### Age-dependent impairments of SPW-R

**Figure 1:**
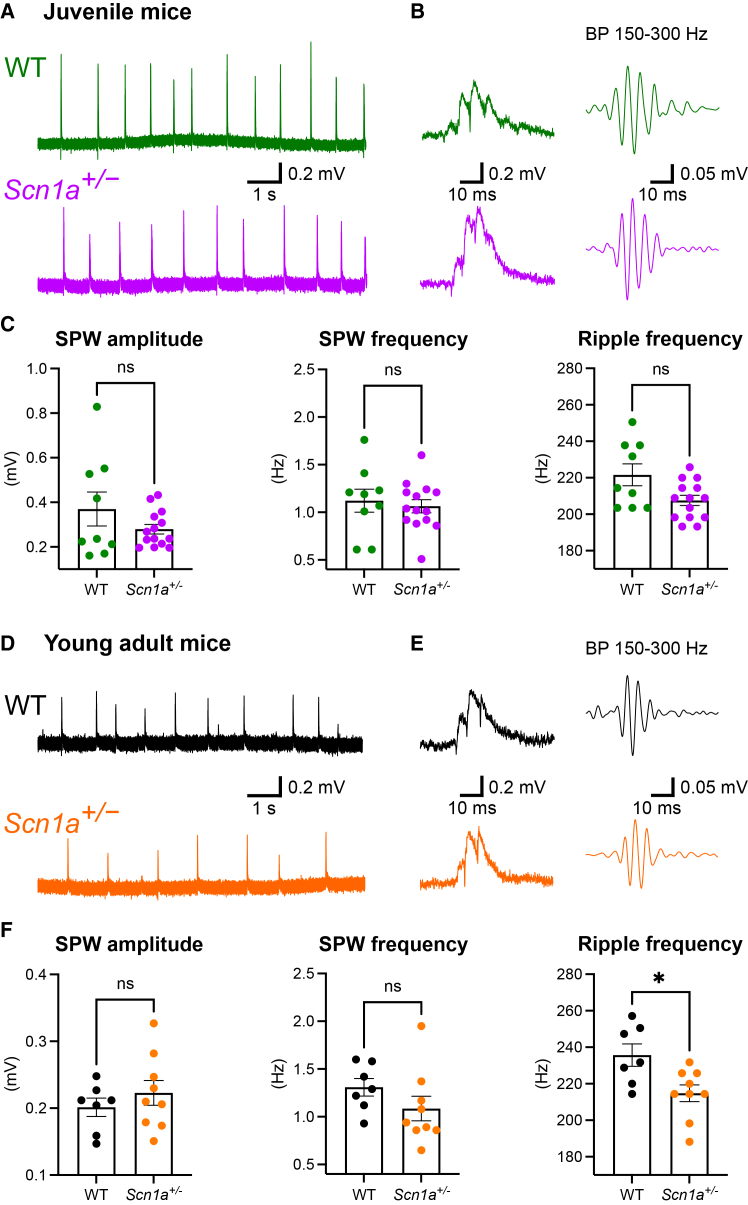
Age-dependent differences in ripple oscillation frequencies between WT and *Scn1a*^+/−^ mice (A) Example traces show spontaneous SPW-R activity. Field potentials were recorded in the pyramidal cell layer of hippocampal area CA1. Data were collected from slices obtained from either juvenile WT (green trace, top) or *Scn1a*^+/−^ mice (magenta trace, bottom). (B) Example traces for SPWs with superimposed ripple oscillations at an expanded timescale (left) and respective band-pass (BP) filtered signals within a frequency range of 150–300 Hz (right). (C) Summary plot for sharp wave amplitudes (left), frequencies of SPW (middle), and ripple oscillation frequencies (right). (D) Example traces from slices obtained from either young adult WT (black trace, top) or *Scn1a*^+/−^ mice (orange trace, bottom). (E) Example traces for SPWs with superimposed ripple oscillations at an expanded timescale (left) and respective band-pass (BP) filtered signals within a frequency range of 150–300 Hz (right). (F) Summary plot for sharp wave amplitudes (left), frequencies of SPW (middle), and ripple oscillation frequencies (right). For (C), summary data from *n* = 9 WT and *n* = 14 *Scn1a*^*+/−*^ mice aged P19-22. For (F), summary data from *n* = 7 WT and *n* = 9 *Scn1a*^*+/−*^ mice aged P30-37. not significant (ns) = *p* > 0.05, ∗ = *p* < 0.05, Welch’s *t* test. Data are represented as mean ± SEM.

To study the effects of a reduced expression of Na_V_1.1 channels on interneuron-dependent network oscillations, we compared spontaneously occurring SPW-R in area CA1 of the hippocampus in slices from WT mice with the activity in slices from *Scn1a*^+/−^ heterozygous knockout mice (Figures 1 and S1). The amplitude and frequency of generation (occurrence) of sharp waves (SPWs) were comparable between juvenile *Scn1a*^+/−^ and WT mice (Figures 1A and 1C; SPW amplitude 0.370 ± 0.076 mV for *n* = 9 WT mice vs. 0.279 ± 0.021 mV for *n* = 14 *Scn1a*^+/−^ mice, *p* = 0.282; SPW frequency 1.12 ± 0.12 Hz WT vs. 1.06 ± 0.07 Hz *Scn1a*^+/−^, *p* = 0.686). We then focused on the ripple oscillations that are occurring as fast superimposed oscillations (Figure 1B) during the majority of detected SPWs, as they are known to depend on the fast synchronized firing of PV-INs and their rhythmic inhibition of surrounding cells.^20^ The analysis of ripples showed no detectable difference in frequency between WT and *Scn1a*^+/−^ mice for the juvenile group, suggesting comparable temporal synchronization of inhibition among PV-INs (Figure 1C; Leading ripple frequency 221.59 ± 6.01 Hz WT vs. 207.58 ± 2.80 Hz *Scn1a*^+/−^, *p* = 0.057).

To see whether the previously described ripple oscillation deficits *in vivo*^12^ may appear only later in development, we performed recordings in slices from young adult mice that survived beyond the time window of high mortality. Recordings in CA1 from *Scn1a*^+/−^ mice again showed SPW-R activity comparable to WT mice (Figures 1D–1F; SPW amplitude 0.201 ± 0.014 mV for *n* = 7 WT mice vs. 0.223 ± 0.019 mV for *n* = 9 *Scn1a*^+/−^ mice, *p* = 0.367; SPW frequency 1.31 ± 0.09 Hz WT vs. 1.09 ± 0.13 Hz *Scn1a*^+/−^, *p* = 0.181). In contrast to juvenile mice, and in line with data from Cheah et al.^12^ the analysis of ripples revealed ripple frequencies for surviving *Scn1a*^+/−^ mice that were, on average, ∼21 Hz lower compared to WT mice (Figure 1F; Leading ripple frequency 235.69 ± 6.12 Hz WT vs. 214.78 ± 4.62 Hz *Scn1a*^+/−^, *p* = 0.019).

### SPW-R-evoked IPSCs in CA1 pyramidal cells

**Figure 2:**
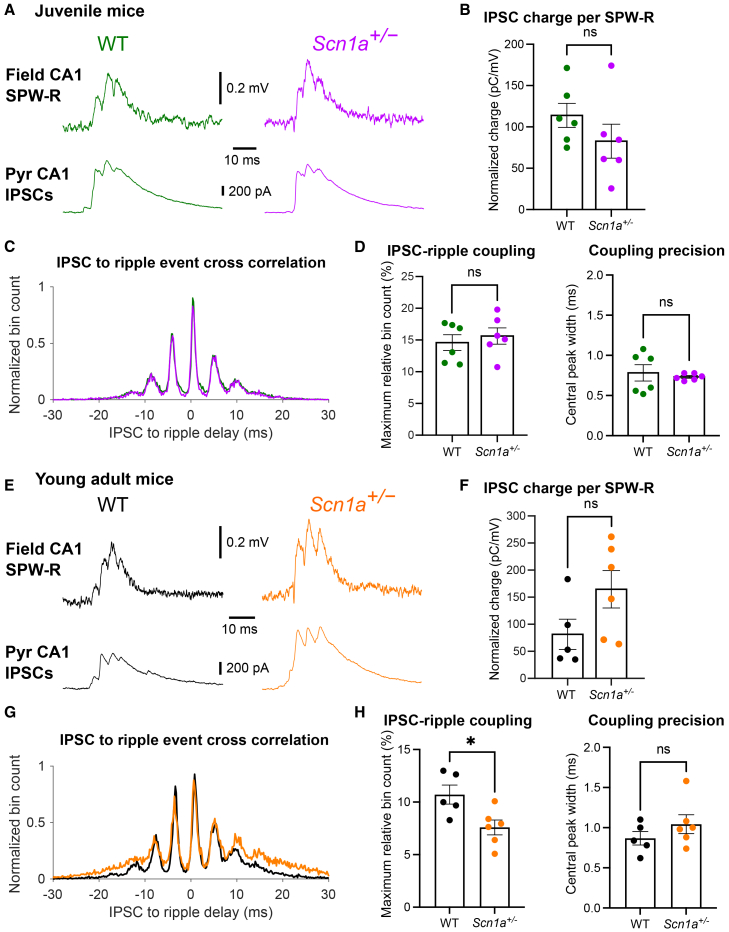
Weaker coupling of IPSCs in CA1 pyramidal cells to local ripple oscillations in slices from young adult *Scn1a*^*+/−*^ mice (A) Representative traces of field (upper traces) and whole-cell voltage clamp (0 mV holding potential) recordings (lower traces) from slices from a juvenile WT (green, left) and *Scn1a*^*+/−*^ (magenta, right) mouse showing SPW-evoked IPSC trains. (B) Summary graphs of normalized SPW-evoked charge values calculated from IPSC trains in pyramidal cells representing the median area under the curve of IPSC trains divided by the respective median SPW amplitude. (C) Mean distributions of normalized event cross-correlations calculated for delays between IPSCs to ripple troughs per SPW-R. (D) Summary graphs for coupling and coupling precision quantifying the central peaks in individual event cross correlations. (E) Representative traces of field (upper traces) and whole-cell voltage clamp (0 mV holding potential) recordings (lower traces) from slices from a young adult WT (black, left) and *Scn1a*^*+/−*^ (orange, right) mouse showing SPW-evoked IPSC trains. (F) Summary graphs of normalized SPW-evoked charge values calculated from IPSC trains in pyramidal cells representing the median area under the curve of IPSC trains divided by the respective median SPW amplitude. (G) Mean distributions of normalized event cross-correlations calculated for delays between IPSCs to ripple troughs per SPW-R. (H) Summary graphs for coupling and coupling precision quantifying the central peaks in individual event cross correlations. For (A–D), summary data from *n* = 6 WT mice, *n* = 6 *Scn1a*^*+/−*^ mice aged P19-22. For (E–H), summary data from *n* = 5 WT mice, *n* = 6 *Scn1a*^*+/−*^ mice aged P35-37. ns = *p* > 0.05, ∗ = *p* < 0.05, Welch’s *t* test. Data are represented as mean ± SEM. See also Figure S2.

To study the synaptic currents underlying the ripple oscillations,^18^^,^^20^^,^^27^ we next recorded IPSCs in CA1 pyramidal cells during SPW-R by using whole-cell voltage-clamp recordings at the reversal potential for excitatory postsynaptic currents (0 mV; Figure 2). As previously shown,^18^^,^^20^^,^^27^ SPW-R were accompanied by trains of IPSCs with a similar temporal structure as the ongoing ripple oscillations.

In slices from juvenile mice, we found similar IPSC charge values (“area under the curve”) per SPW-R for both groups. To account for differences in excitatory drive to the local network and the known correlation between SPW amplitude and synaptic input,^27^ we normalized the charge by dividing each value by the respective SPW amplitude (Figures 2A and 2B; Normalized charge 113.82 ± 14.54 pC mV^−1^ for *n* = 6 WT mice vs. 82.64 ± 20.56 pC mV^−1^ for *n* = 6 *Scn1a*^+/−^ mice, *p* = 0.247). Using cross-correlation analyses, we found that individual IPSC onsets during the trains were temporally coupled to ripple troughs in both genotype groups (Figures 2C and 2D; IPSC-ripple coupling (maximum relative bin count) 14.58 ± 1.25% WT vs. 15.62 ± 1.28% *Scn1a*^+/−^, *p* = 0.574; coupling precision (central peak width) 0.78 ± 0.10 ms WT vs. 0.73 ± 0.02 ms *Scn1a*^+/−^, *p* = 0.647).

**Figure 3:**
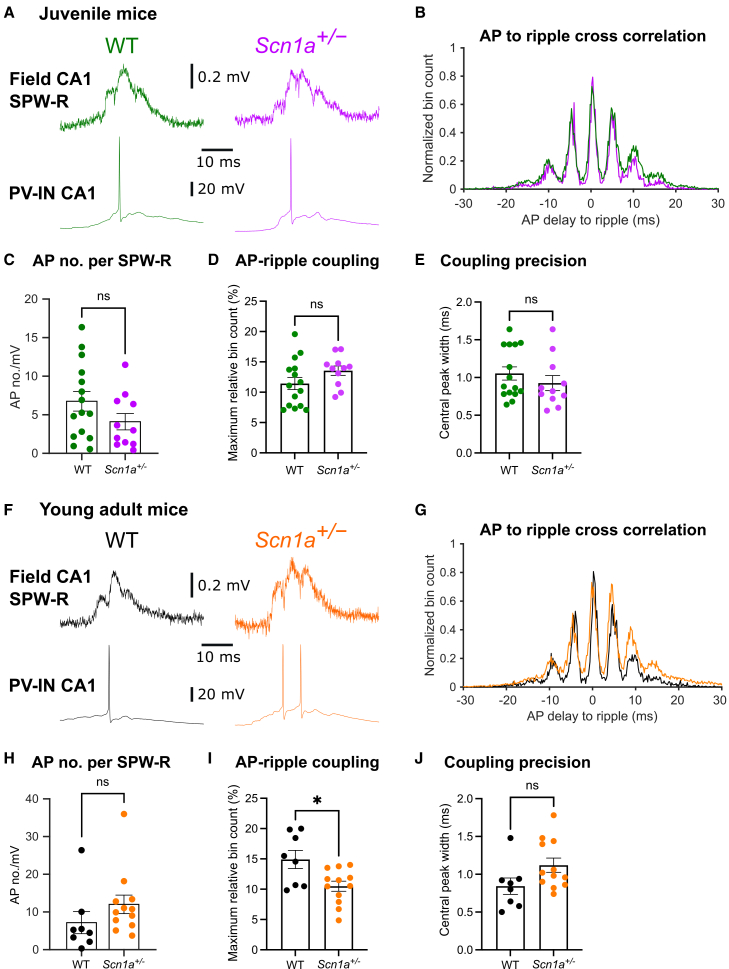
Differential AP coupling to ripple oscillations in PV-INs of WT and *Scn1a*^*+/−*^ mice (A) Representative traces of field potential (upper traces) and whole-cell current-clamp recordings (lower traces) from slices prepared from either juvenile WT (green, left) or *Scn1a*^*+/−*^ (magenta, right) mice showing SPW-R-evoked APs. (B) Mean distribution of normalized event cross-correlations calculated between detected APs and ripple troughs for each SPW-R per mouse for both groups. (C) Summary plot for the number of APs per SPW-R normalized for SPW amplitude. (D) Summary plot for the maximum bin count for AP-to-ripple delays in the respective histogram for each recording. (E) The width (sharpness) of the central peak of the respective histogram for each recording representing coupling precision. (F) Representative traces from slices from either young adult WT (black, left) or *Scn1a*^*+/−*^ (orange, right) mice showing SPW-R-evoked APs. (G) Mean distribution of normalized event cross-correlations calculated between detected APs and ripple troughs for each SPW-R per mouse for both groups. (H) Summary plot for the number of APs per SPW-R divided by SPW amplitude. (I) Summary plot for the maximum bin count for AP to ripple delays in the respective histogram for each recording. (J) The width (sharpness) of the central peak of the respective histogram for each recording representing coupling precision. For (B–E), summary data from *n* = 15 WT and *n* = 11 *Scn1a*^*+/−*^ mice aged P19-22. For (H–J), summary data from *n* = 8 WT and *n* = 12 *Scn1a*^*+/−*^ mice aged P30-37. ns = *p* > 0.05, ∗ = *p* < 0.05, Welch’s *t* test. Data are represented as mean ± SEM. See also Figures S3–S5.

In young adult mice, we found a fraction of pyramidal cells from *Scn1a*^*+/−*^ mice with a large IPSC charge per SPW-R, which would be in agreement with an increased number of APs per SPW-R in PV-INs (Figure 3H), but there was no detectable group difference (Figures 2E and 2F; normalized charge 81.30 ± 27.94 pC mV^−1^ for *n* = 5 WT mice vs. 164.47 ± 34.52 pC mV^−1^ for *n* = 6 *Scn1a*^+/−^ mice, *p* = 0.094). The event cross-correlation between IPSC onsets and ripple troughs revealed a slightly broader mean distribution of delays (Figure 2G). Quantification of the distribution confirmed a lower temporal coupling between IPSC trains and ripple oscillations in *Scn1a*^+/−^ compared to WT mice, while there were no detectable differences in coupling precision (Figures 2G and 2H; IPSC-ripple coupling 10.71 ± 0.90% WT vs. 7.59 ± 0.70% *Scn1a*^+/−^, *p* = 0.026; coupling precision 0.87 ± 0.08 ms WT vs. 1.04 ± 0.12 ms *Scn1a*^+/−^, *p* = 0.257).

As the largely intact synaptic inhibition during SPW-R may appear contradictory to previous reports showing a reduction in AP-dependent spontaneous IPSCs (sIPSCs) in *Scn1a*^*+/−*^ mice,^29^^,^^31^^,^^32^ we also recorded sIPSCs and miniature IPSCs (mIPSCs) from CA1 pyramidal cells in slices without SPW-R (Figure S2). Similarly to previous results, we found a lower frequency of large-amplitude IPSCs in slices from juvenile *Scn1a*^*+/−*^ compared to WT mice, compatible with a lower spontaneous AP firing of PV-IN. Comparison of mIPSCs revealed no difference between groups, indicating a similar number of inhibitory synapses onto pyramidal cells.

### SPW-R-evoked firing in PV-INs

We then studied whether an altered AP firing of PV-INs may be responsible for the ripple oscillation deficits (Figure 1F) and the alterations in the rhythmic coupling between IPSC trains and ripple oscillations (Figures 2G and 2H) we found in young adult but not in juvenile *Scn1a*^+/−^ mice. For this, we analyzed SPW-R-evoked APs in PV-INs from the same age and genotype groups using whole-cell patch-clamp recordings from somata of PV-INs in CA1 within ∼100 μm from the field potential recording pipette (Figure 3). PV-INs are known to receive trains of excitatory and inhibitory synaptic inputs generating APs that are phase-locked to the ripple oscillations during SPW-R.^20^^,^^33^^,^^34^^,^^35^

In slices from juvenile mice, we observed SPW-R-evoked AP firing in PV-INs at similar levels in both WT and *Scn1a*^+/−^ mice, which were highly correlated to individual ripple cycles (Figures 3A and 3B). There were no detectable group differences in the activation of PV-INs quantified by the average number of evoked APs and the excitatory postsynaptic current (EPSC) charge per SPW-R (Figures S3B–S3D; AP no. 1.76 ± 0.27 for *n* = 15 WT mice vs. 1.06 ± 0.29 for *n* = 11 *Scn1a*^+/−^ mice, *p* = 0.087; EPSC charge 26.06 ± 3.51 pC for *n* = 10 WT mice vs. 23.01 ± 4.20 pC for *n* = 11 *Scn1a*^+/−^ mice, *p* = 0.585), as well as when normalized with the SPW amplitude as measure for the excitatory drive to the local network^27^ (Figure 3C; AP no. normalized with SPW amplitude 6.74 ± 1.27 mV^−1^ for *n* = 15 WT mice vs. 4.10 ± 1.06 mV^−1^ for *n* = 11 *Scn1a*^+/−^ mice, *p* = 0.123). There were also no differences in the rhythmic correlation between automatically detected APs and individual ripple troughs quantified by the event cross-correlation analysis (Figures 3B, 3D, and 3E; AP-ripple coupling 11.44 ± 1.00% WT vs. 13.56 ± 0.77% *Scn1a*^+/−^, *p* = 0.106; coupling precision 1.05 ± 0.09 ms WT vs. 0.93 ± 0.10 ms *Scn1a*^+/−^, *p* = 0.348).

Recordings in slices from young adult mice (Figures 3F–3J), in turn, revealed a stronger activation of PV-INs per SPW-R for *Scn1a*^*+/−*^ mice (Figures S3E–S3H; AP no. 1.16 ± 0.42 for *n* = 8 WT mice vs. 2.86 ± 0.46 for *n* = 12 *Scn1a*^+/−^ mice, *p* = 0.014; EPSC charge 13.83 ± 1.33 pC for *n* = 7 WT mice vs. 22.99 ± 2.25 pC for *n* = 11 *Scn1a*^+/−^ mice, *p* = 0.003), while the AP number did not differ between groups after normalization with the overall excitatory drive represented by SPW amplitude (Figure 3H; AP no. normalized with SPW amplitude 7.16 ± 2.93 mV^−1^ WT mice vs. 12.01 ± 2.45 mV^−1^ *Scn1a*^+/^, *p* = 0.223). The event cross-correlation analysis between APs in PV-INs and ripple troughs in slices from *Scn1a*^+/−^ mice revealed a weaker coupling compared to WT mice (Figure 3I; Peak correlation 14.91 ± 1.50% WT vs. 10.49 ± 0.83% *Scn1a*^+/−^, *p* = 0.025), while there were no detectable group difference in the coupling precision (Figure 3J; Coupling precision 0.84 ± 0.11 ms WT vs. 1.12 ± 0.09 ms *Scn1a*^+/−^, *p* = 0.070). Together, these data indicate an increased but less synchronized AP firing in young adult *Scn1a*^*+/−*^ compared to WT mice, which was not detectable at the juvenile stage.

### SPW-R-evoked IPSCs in PV-INs

**Figure 4:**
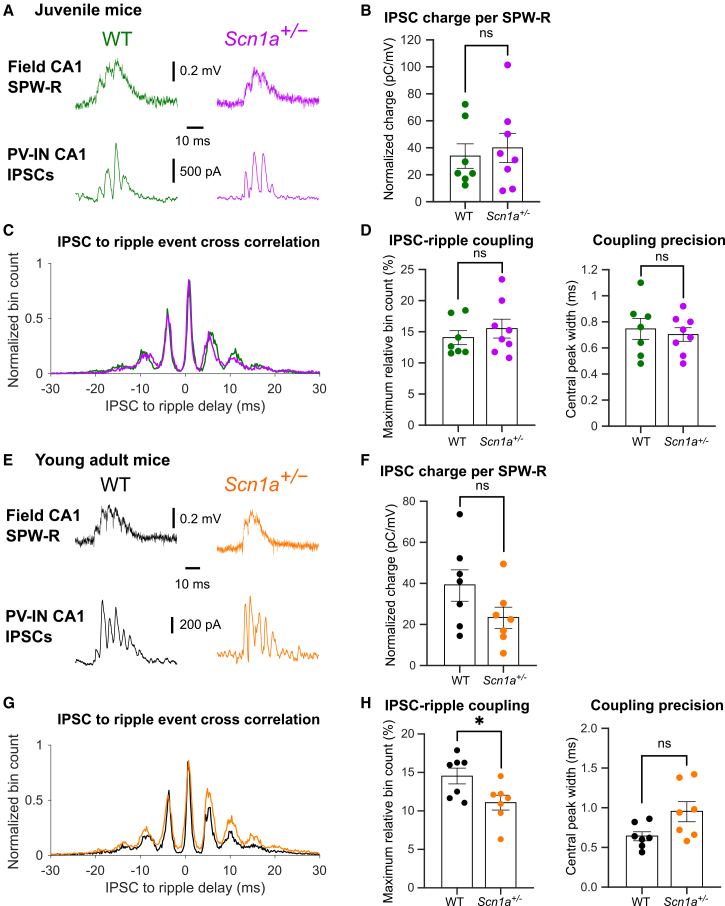
Weaker coupling of IPSCs in CA1 PV-INs to local ripple oscillations in slices from young adult *Scn1a*^*+/−*^ mice (A) Representative traces of field (upper traces) and whole-cell voltage clamp (0 mV holding potential) recordings (lower traces) from slices from a juvenile WT (green, left) and *Scn1a*^*+/−*^ (magenta, right) mouse showing SPW-R-evoked IPSC trains. (B) Summary graphs of normalized SPW-R-evoked charge values calculated from IPSC trains in PV-INs representing the median area under the curve of IPSC trains divided by the respective median SPW amplitude. (C) Mean distributions of normalized event cross-correlations calculated for delays between IPSCs to ripple troughs per SPW-R. (D) Summary graphs for coupling and coupling precision quantifying the central peaks in individual event cross correlations. (E) Representative traces of field (upper traces) and whole-cell voltage clamp (0 mV holding potential) recordings (lower traces) from slices from a young adult WT (black, left) and *Scn1a*^*+/−*^ (orange, right) mouse showing SPW-R-evoked IPSC trains. (F) Summary graphs of normalized SPW-R-evoked charge values calculated from IPSC trains in pyramidal cells representing the median area under the curve of IPSC trains divided by the respective median SPW amplitude. (G) Mean distributions of normalized event cross-correlations calculated for delays between IPSCs to ripple troughs per SPW-R. (H) Summary graphs for coupling and coupling precision quantifying the central peaks in individual event cross correlations. For (A–D), summary data from *n* = 7 WT mice, *n* = 8 *Scn1a*^*+/−*^ mice aged P19-22. For (E–H), summary data from *n* = 7 WT mice, *n* = 7 *Scn1a*^*+/−*^ mice aged P30-37. ns = *p* > 0.05, ∗ = *p* < 0.05, Welch’s *t* test. Data are represented as mean ± SEM.

To test whether changes in the mutual inhibition between PV-INs may contribute to the difference in AP to SPW-R coupling (Figure 3I), we analyzed SPW-R-evoked IPSCs in the same way as for pyramidal cells. In slices from juvenile mice, we again found similar levels of SPW-R-evoked IPSC trains in *Scn1a*^+/−^ compared to WT mice and no differences in the coupling between individual IPSCs and ripple cycles (Figures 4A–4D; Normalized charge 33.84 ± 9.07 pC mV^−1^ for *n* = 7 WT mice vs. 39.91 ± 10.82 pC mV^−1^ for *n* = 8 *Scn1a*^+/−^ mice, *p* = 0.247; IPSC-ripple coupling 14.05 ± 1.13% WT vs. 15.50 ± 1.51% *Scn1a*^+/−^, *p* = 0.458; coupling precision 0.75 ± 0.08 ms WT vs. 0.70 ± 0.05 ms *Scn1a*^+/−^, *p* = 0.661).

In slices from young adult mice, we also found no group differences in the IPSC charge values (Figures 4E and 4F; Normalized charge 39.26 ± 7.71 pC mV^−1^ for *n* = 7 WT mice vs. 23.39 ± 5.26 pC mV^−1^ for *n* = 7 *Scn1a*^+/−^ mice, *p* = 0.118). In turn, the event cross-correlation between IPSC onsets and ripple troughs revealed a slightly broader mean distribution of delays (Figure 4G), similar to data from pyramidal cells (Figure 2G). Quantification of the distribution confirmed a lower temporal coupling between IPSC trains in PV-INs and ripple oscillations in *Scn1a*^+/−^ compared to WT mice, while there were no detectable differences in coupling precision (Figure 4H; IPSC-ripple coupling 14.51 ± 1.02% WT vs. 11.07 ± 0.96% *Scn1a*^+/−^, *p* = 0.030; coupling precision 0.64 ± 0.06 ms WT vs. 0.95 ± 0.13 ms *Scn1a*^+/−^, *p* = 0.055). These data are consistent with those about the inhibition onto pyramidal cells and the changes in rhythmic AP firing of young adult *Scn1a*^*+/−*^ mice.

### Age-dependent changes of AP waveforms in PV-INs of *Scn1a*^*+/−*^ mice

**Figure 5:**
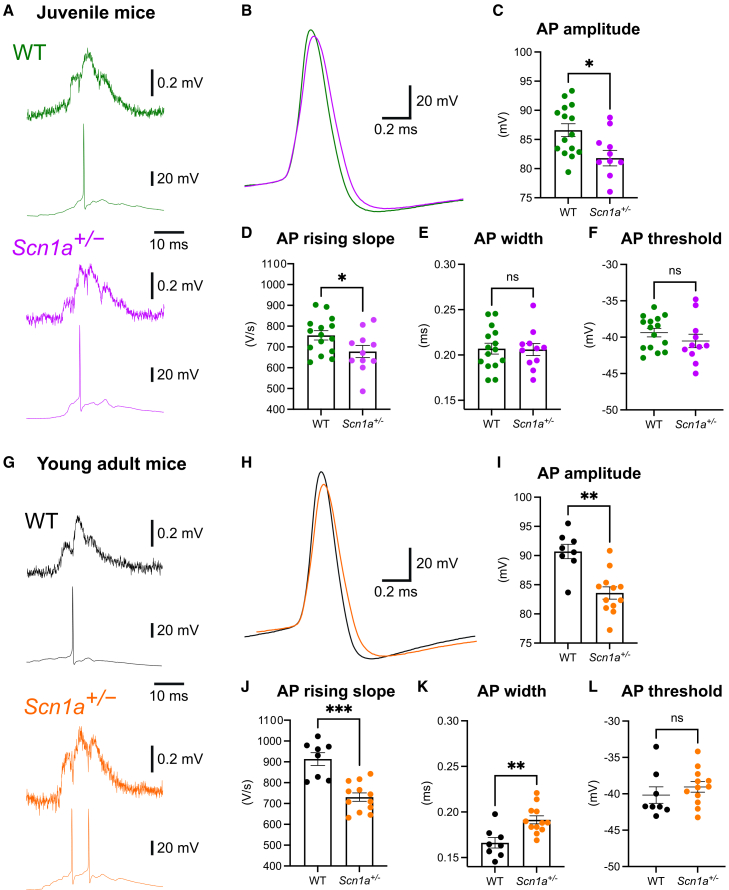
APs during SPW-R show age-dependent deficits in PV-IN from *Scn1a*^+/−^ mice (A) Spontaneous SPW-R field activity and SPW-R-evoked AP firing in slices from juvenile WT (green traces) and *Scn1a*^*+/−*^ (magenta traces) mice. (B) Overlay of SPW-R-evoked APs recorded from either a PV-IN from a juvenile WT mouse or a *Scn1a*^+/−^ mouse. (C–F) Summary plots for AP amplitude, AP rising slope, AP width, and AP threshold. (G) Spontaneous SPW-R field activity and SPW-R-evoked AP firing in slices from young adult WT (black traces) and *Scn1a*^*+/−*^ (orange traces) mice. (H) Overlay of SPW-R-evoked APs recorded from either a PV-IN from a young adult WT mouse or a *Scn1a*^+/−^ mouse. (I–L) Summary plots for AP amplitude, AP rising slope, AP width, and AP threshold. For (C–F), summary data from *n* = 15 WT mice, *n* = 11 *Scn1a*^+/−^ mice aged P19-22. For (I–L), summary data from *n* = 8 WT mice, *n* = 12 *Scn1a*^+/−^ mice aged P30-37. ns = *p* > 0.05, ∗ = *p* < 0.05, ∗∗ = *p* < 0.01, ∗∗∗ = *p* < 0.001, Welch’s *t* test. Data are represented as mean ± SEM. See also Figure S4 and Table S1.

To study the mechanisms linking the *Scn1a* haploinsufficiency to the observed changes in AP timing and synchronization of synaptic inhibition provided by PV-INs in an age-dependent manner, we analyzed the AP waveforms of SPW-R-evoked APs to identify reduced Na_V_ channel activity and possible consequences in the timing of APs (Figure 5; Table S1). APs in PV-INs from juvenile *Scn1a*^+/−^ mice had slightly smaller amplitudes (by ∼5 mV) and lower rising slopes compared to WT mice but did not differ in width or threshold (Figures 5A–5F; AP amplitude 86.60 ± 1.11 mV for *n* = 15 WT mice vs. 81.80 ± 1.33 mV for *n* = 11 *Scn1a*^+/−^ mice, *p* = 0.011; rising slope 755.77 ± 22.28 V/s WT vs. 677.92 ± 29.17 V/s *Scn1a*^+/−^, *p* = 0.047; width 0.207 ± 0.006 ms WT vs. 0.206 ± 0.007 ms *Scn1a*^+/−^, *p* = 0.916; threshold −39.38 ± 0.58 mV WT vs. −40.52 ± 0.93 mV *Scn1a*^+/−^, *p* = 0.311).

In young adults, SPW-R-evoked APs of PV-INs in slices prepared from *Scn1a*^+/−^ mice were also slightly lower in amplitude (by ∼7 mV) and had even lower rising slopes compared to WT mice. However, in contrast to juvenile PV-INs, the APs were broader when compared to WT mice, while the AP thresholds remained unchanged (Figures 5H–5L; AP amplitude 90.70 ± 1.23 mV for *n* = 8 WT mice vs. 83.59 ± 1.05 mV for *n* = 12 *Scn1a*^+/−^ mice, *p* < 0.001; rising slope 913.41 ± 30.60 V/s WT vs. 730.70 ± 20.26 V/s *Scn1a*^+/−^, *p* < 0.001; width 0.166 ± 0.006 ms WT vs. 0.191 ± 0.004 ms *Scn1a*^+/−^, *p* = 0.004; threshold −40.17 ± 1.14 mV WT vs. −39.06 ± 0.72 mV *Scn1a*^+/−^, *p* = 0.423).

These data also revealed a more pronounced age-dependent increase in AP amplitude and rising slope and a reduction of AP width for *Scn1a*^+/−^ compared to WT mice (difference between young adult and juvenile WT mice: AP amplitude +4.1 mV, *p* = 0.024, rising slope +157.6 V/s, *p* < 0.001, width −0.040 ms, *p* < 0.001; differences for *Scn1a*^+/−^ mice: AP amplitude +1.8 mV, rising slope +52.8 V/s, width −0.015 ms, *p* > 0.05 for all comparisons of *Scn1a*^+/−^ data).

During these experiments with ongoing SPW-R oscillations, we also performed somatic current injections to analyze the passive and active properties of the cells (Figure S4; Table S2). In contrast to previous reports from neocortical and hippocampal fast-spiking interneurons,^8^^,^^24^^,^^29^^,^^36^ high-frequency trains of APs evoked by submaximal 1-*s*-current pulses showed no impairments of the fast-spiking phenotype in PV-INs in our slices from *Scn1a*^+/−^ mice compared to slices from WT mice in both age groups (Figures S4A–S4C and S4E–S4G).

Similarly to the findings from SPW-associated firing, APs of PV-INs in slices from juvenile *Scn1a*^+/−^ mice were of lower amplitudes and rising slope, while showing no difference in width compared to WT mice or in other electrophysiological parameters (Figure S4D; Table S2).

In slices of surviving young adult *Scn1a*^+/−^ mice, APs of PV-INs were not only of lower amplitude and rising slope but were also broader compared to WT mice (Figure S4H; Table S2). For comparison, we also recorded current-induced APs in CA1 pyramidal cells from young adult mice. Consistent with previous reports,^7^^,^^37^^,^^38^ we found no differences in AP waveforms in these recordings (Figure S6).

To test whether the previously reported fast-spiking deficits in GABAergic interneurons of CA1^29^ may be masked by the ongoing SPW-R-induced synaptic and firing activity, we repeated the recordings of input-output relationships in slices from conventional submerged beaker incubation in a standard recording chamber lacking high-flow double perfusion. In these slices, strong current injections revealed a lower maximum firing frequency and stronger use-dependent AP amplitude attenuation at high frequencies for PV-INs from juvenile *Scn1a*^+/−^ compared to WT mice (Figures S5A and S5B; Maximum AP frequency 426.33 ± 14.61 Hz for *n* = 9 cells from 4 WT mice vs. 367.53 ± 9.90 Hz for *n* = 13 cells from 5 *Scn1a*^+/−^ mice, *p* = 0.007). In recordings from PV-INs from young adult mice, in turn, there were no detectable differences in maximum firing frequency between groups, but cells from *Scn1a*^+/−^ mice also showed strong use-dependent AP amplitude attenuations at strong current injections (Figures S5C and S5D; Maximum AP frequency 426.38 ± 12.95 Hz for *n* = 13 cells from 5 WT mice vs. 410.83 ± 16.02 Hz for *n* = 12 cells from 5 *Scn1a*^+/−^ mice, *p* = 0.459). Together, these data suggest a similar age-dependent expression of fast-spiking deficits in PV-INs as reported previously.^8^^,^^10^^,^^24^^,^^29^

### TTX differentially modulates APs in young adult WT vs. *Scn1a*^*+/−*^ mice

**Figure 6:**
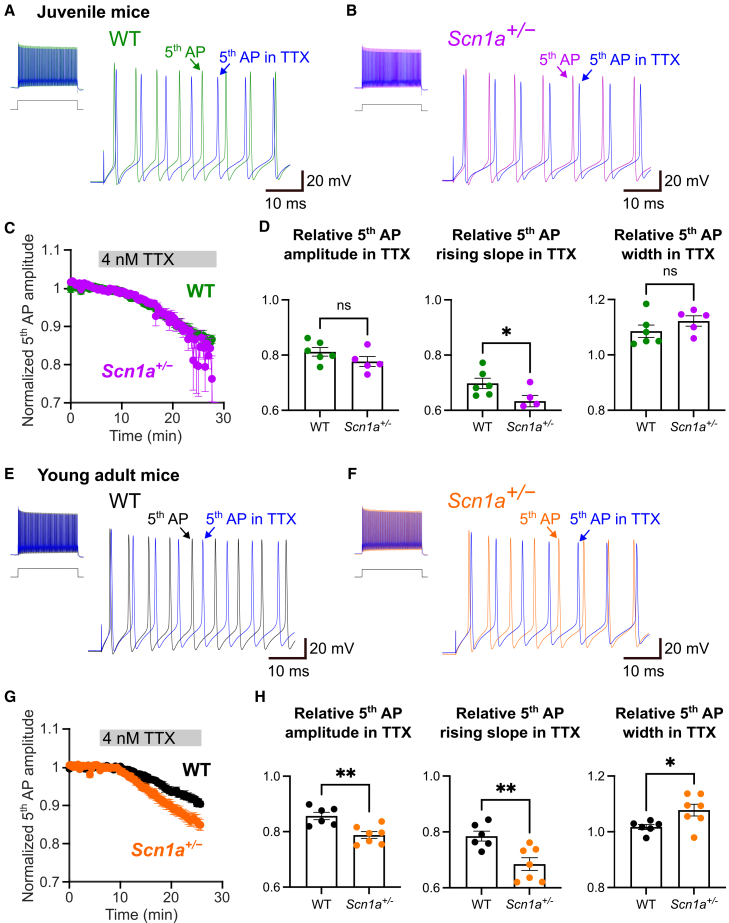
A low dose of TTX induces differential effects in young adult WT vs. *Scn1a*^+/−^ mice (A) Overlay of current-evoked AP trains (inset, left) and the first 50 ms of the trains (right) recorded in a PV-IN from a juvenile WT mouse in control conditions (green trace) and after 20 min in 4 nM TTX (blue trace). (B) Similar to (A), but an overlay of data from a juvenile *Scn1a*^*+/−*^ mouse. (C) Mean ± SEM of normalized amplitudes of the 5^th^ APs for juvenile WT and *Scn1a*^*+/−*^ mice during application of 4 nM TTX. (D) Relative TTX-induced changes in AP amplitude, AP rising slope, and AP width after 20 min of 4 nM TTX bath application. (E) Overlay of AP trains recorded in a PV-IN from a young adult WT mouse in control conditions (black trace) and after 20 min in 4 nM TTX (blue trace). (F) Similar to (E), but an overlay of data from a young adult *Scn1a*^*+/−*^ mouse. (G) Mean ± SEM of normalized amplitudes of the 5^th^ APs for young adult WT and *Scn1a*^+/−^ mice during the application of 4 nM TTX. (H) Relative TTX-induced changes in AP amplitude, AP rising slope, and AP width after 20 min of 4 nM TTX bath application. For (C and D), summary data from *n* = 6 WT mice, *n* = 5 *Scn1a*^*+/−*^ mice aged P20-22. For (G and H), summary data from *n* = 6 WT mice, *n* = 7 *Scn1a*^+/−^ mice aged P31-36. ns = *p* > 0.05, ∗ = *p* < 0.05, ∗∗ = *p* < 0.01, Welch’s *t* test. Data are represented as mean ± SEM.

SPW-R-evoked firing of PV-INs showed AP deficits compatible with a reduction in the available Na_V_ channel pool. According to this hypothesis, APs in PV-INs from *Scn1a*^+/−^ mice are thus expected to be more susceptible to a further decrease in the number of activatable Na_V_ channels by a partial block using TTX.^39^ To test this, we used square-pulse current injection to evoke comparable numbers of somatically recorded APs in WT or *Scn1a*^+/−^ mice (100–130 Hz firing rates, Figures 6A, 6B, 6E, and 6F) in combination with the application of a low dose of TTX.^24^^,^^39^ We determined a concentration of 4 nM TTX, which is close to the reported IC50 for Na_V_1.1,^40^ as the minimal dose leading to a notable reduction of the AP number in slices of WT mice within 30 min under our experimental condition. During the application of 4 nM TTX, AP trains showed reduced AP frequencies and progressively smaller and slower APs (Figure 6). These progressive changes in AP waveforms during the early phase of the train are compatible with a use-dependent reduction of available Na_V_ channels during repetitive firing with incomplete recovery from inactivation (Figures 6A, 6B, 6E, and 6F). For comparing genotype groups, we chose to analyze the 5^th^ AP of each train. This represents a compromise between the use-dependent effect on the APs and the relevance for network activity such as SPW-R, which induce only a limited number of APs in PV-INs.

In agreement with our previous findings from juvenile mice, there were only small differences in the TTX-induced effects on the AP waveform between juvenile WT and *Scn1a*^+/−^ mice (Figure 6C). Nevertheless, after 20 min of 4 nM TTX bath application, we observed a stronger reduction of the AP rising slope in *Scn1a*^+/−^ mice, while there were no detectable differences in other AP parameters (Figure 6D; Relative 5^th^ AP amplitude 0.81 ± 0.02 for *n* = 6 WT mice vs. 0.78 ± 0.02 for *n* = 5 *Scn1a*^+/−^ mice, *p* = 0.174; relative rising slope 0.70 ± 0.02 WT vs. 0.63 ± 0.02 *Scn1a*^+/−^, *p* = 0.047; relative width 1.09 ± 0.02 WT vs. 1.12 ± 0.02 *Scn1a*^+/−^, *p* = 0.239).

For young adult mice (Figures 6E–6H), the average amplitude of the 5^th^ AP decreased more strongly for *Scn1a*^+/−^ than for WT mice after 20 min of TTX application (Figures 6G and 6H; Reduction by about 14% for WT mice and 20% for *Scn1a*^+/−^ mice; amplitude for 5^th^ AP decreased to 0.86 ± 0.01 compared to baseline values before TTX in *n* = 6 WT mice vs. 0.79 ± 0.01 for *n* = 7 *Scn1a*^+/−^ mice, *p* = 0.003). In agreement with a reduced Na_V_ channel pool in PV-INs of young adult *Scn1a*^+/−^ mice, reductions in AP rising slopes and an increased AP width were more pronounced in *Scn1a*^+/−^ compared to WT mice (Figure 6H; Relative rising slope for 5^th^ AP 0.79 ± 0.02 for *n* = 6 WT mice, vs. 0.68 ± 0.02 for *n* = 7 *Scn1a*^+/−^ mice, *p* = 0.005; relative width 1.02 ± 0.01 WT vs. 1.08 ± 0.02 *Scn1a*^+/−^, *p* = 0.029). These data suggest an age-dependent decrease in the Na_V_ channel pool in *Scn1a*^+/−^ mice, as shown by the increased added effects on the AP waveforms caused by a combination of *Scn1a* haploinsufficiency and application of TTX in hippocampal PV-INs.

### Axonal APs of PV-INs are impaired in young adult *Scn1a*^*+/−*^ mice

**Figure 7:**
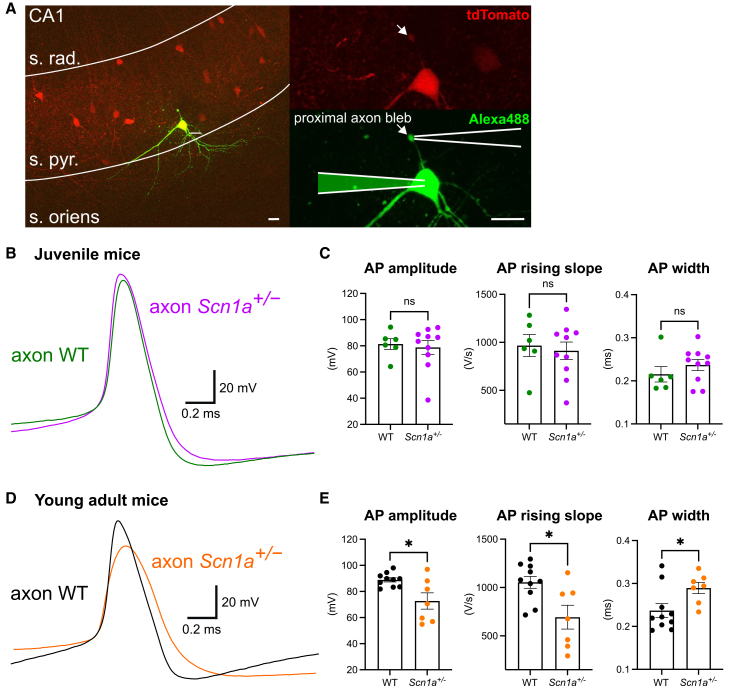
Axonal recordings from PV-INs confirm an age-dependent AP deficit (A) Illustration of whole-cell current clamp recordings from the proximal axon of PV-INs. Left, combined two-photon image of tdTomato and Alexa 488 identifying the patched PV-IN in stratum pyramidale (s. pyr.). Right, individual imaging channels at higher magnification. Recording pipettes are added for illustrative purposes only. Scale bars represent 20 μm. s. rad., stratum radiatum; s. oriens, stratum oriens. (B) Overlay of axonal APs induced by somatic current injection to either a PV-IN from a juvenile WT (green trace) or *Scn1a*^+/−^ mouse (magenta trace). (C) Summary plots for AP amplitude, AP rising slope, and AP width for juvenile WT and *Scn1a*^+/−^ mice. (D) Overlay of axonal APs induced by somatic current injection to either a PV-IN from a young adult WT (black trace) or *Scn1a*^+/−^ mouse (orange trace). (E) Summary plots for AP amplitude, AP rising slope, and AP width for young adult WT and *Scn1a*^+/−^ mice. For (C), summary data from *n* = 6 WT mice, *n* = 10 *Scn1a*^+/−^ mice aged P19-23. For (E), summary data from *n* = 10 WT mice, *n* = 7 *Scn1a*^+/−^ mice aged P30-34. ns = *p* > 0.05, ∗ = *p* < 0.05, Mann-Whitney U test. Data are represented as mean ± SEM.

Na_V_ channels are highly expressed in the axon, and particularly at the axon initial segment (AIS), where APs are typically initiated. Based on this notion, we next studied axon-specific AP deficits close to the AP initiation site where differences in Na_V_1.1 expression may potentially lead to stronger consequences for AP generation or the AP waveforms. For this, we directly recorded APs in the proximal axon (20–50 μm distance from the soma) of PV-INs using whole-cell current clamp (Figure 7), and APs were elicited by somatic current injections through a second pipette at the soma. In contrast to data from somatic recordings (Figures 5 and S4), recordings from axons in slices from juvenile mice revealed no differences between *Scn1a*^+/−^ and WT mice in terms of AP waveform, indicating comparable Na_V_ channel function in the proximal axon (Figures 7B and 7C; AP amplitude 81.37 ± 4.07 mV for *n* = 6 WT mice vs. 78.82 ± 5.25 mV for *n* = 10 *Scn1a*^+/−^ mice, *p* = 0.707; rising slope 966.41 ± 114.39 V/s WT vs. 912.41 ± 91.12 V/s *Scn1a*^+/−^, *p* = 0.719; width 0.215 ± 0.018 ms WT vs. 0.237 ± 0.013 ms *Scn1a*^+/−^, *p* = 0.368, Mann-Whitney U test). Thus, the slight decrease in AP amplitude observed in somatic recordings of SPW-R-evoked and current-evoked APs was not present in the axonal compartment.

While axonal APs from juvenile animals were similar between both groups, PV-IN axons from young adult *Scn1a*^+/−^ mice generated smaller, slower, and broader APs compared to axons in slices from WT mice (Figures 7D and 7E; AP amplitude 88.91 ± 1.63 mV for *n* = 10 WT mice vs. 72.70 ± 6.27 mV for *n* = 7 *Scn1a*^+/−^ mice, *p* = 0.042; rising slope 1053.58 ± 61.59 V/s WT vs. 693.35 ± 123.13 V/s *Scn1a*^+/−^, *p* = 0.028; width 0.237 ± 0.016 ms WT vs. 0.289 ± 0.013 ms *Scn1a*^+/−^, *p* = 0.023). This is consistent with our results from somatic recordings (Figures 5 and S4) but reveals a stronger difference in AP amplitude between the two genotype groups at young adult ages (∼16 mV for axonal, ∼7 mV for somatic APs).

## Discussion

DS is associated with cognitive disabilities, which may result from general neural circuit dysfunctions.^4^^,^^5^ In our study, we found that SPW-R, a hippocampal network activity pattern that is known to be involved in memory consolidation,^13^^,^^14^^,^^15^^,^^16^ is impaired in young adult but not in juvenile *Scn1a*^*+/*−^ mice. During SPW-R, a reduced ripple frequency was accompanied by changes in rhythmic AP firing in PV-INs and in their synaptic output to pyramidal cells, as well as changes in the somatic and axonal AP waveforms. Also, when using a partial block of Na_V_ channels by TTX, APs in PV-INs from young adult *Scn1a*^*+/−*^ mice were more strongly affected than those from WT mice. Collectively, these changes are consistent with a stronger reduction of the active Na_V_ channel pool toward older ages and a synchronization deficit among PV-INs in area CA1 of the hippocampus of young adult *Scn1a*^*+/−*^ mice. These data may contribute to a better understanding of the known long-term cognitive impairments in older patients with DS and are likely less related to epileptic seizure generation, which tends to improve after the first years of life.

### SPW-R deficits and cognitive impairments in DS

SPW-R are important for memory acquisition and retrieval in rodents,^13^^,^^14^^,^^15^^,^^16^^,^^41^ and impaired SPW-R activity was found in *in vivo* studies on DS mice.^12^^,^^42^ Despite their prominent role in the hippocampus of rodents, it has also been shown in humans that ripple oscillations positively correlate with cognitive performance.^43^^,^^44^ In addition to the well-studied general association between epilepsy (including DS) and cognitive deficits,^4^^,^^5^^,^^45^ it was also shown in mice that a reduced expression of *Scn1a* impaired spatial memory performance in behavioral test.^29^ We propose that the study of the mechanisms underlying ripple oscillation deficits may thus help to link cellular deficits associated with (genetic) epilepsies mostly studied *in vitro* to *in vivo* dysfunctions possibly causing cognitive impairments.

### SPW-R in juvenile and young adult *Scn1a*^*+/−*^ mice

Our *in vitro* model with optimized storage conditions for hippocampal brain slices^17^^,^^19^^,^^20^^,^^26^^,^^46^ allows the investigation of SPW-R and associated cellular activity down to axonal resolution. We used this approach to investigate the hypothesis that PV-IN-dependent network activity is more strongly impaired in animals before mortality onset (∼P24) compared to older animals, as suggested by data showing that PV-INs in the neocortex and hippocampus showed the strongest fast-spiking deficits during the early age-window (∼P18-24).^8^^,^^24^^,^^29^ In stark contrast, we found no difference in the generation of SPW-R and the frequency of ripple oscillations between *Scn1a*^+/−^ and WT mice at the ages between P19-23 in our hippocampal slices, although we also replicated the transient fast-spiking deficit at this age in slices from *Scn1a*^+/−^ mice without SPW-R oscillations. While the time-course of fast-spiking deficits may suggest a decreasing impact of the Na_V_ channel deficiency with increasing age,^8^^,^^24^ our data revealed an opposite time course for changes in ripple oscillation frequencies during SPW-R, which was selective for young adult (P30-37) *Scn1a*^+/−^ mice. This may suggest a stronger disturbance of mechanisms underlying ripple oscillations with increasing age, which we then linked to changes in AP waveforms. These findings may thus be relevant for surviving mice as a model for persisting deficits at older ages,^24^ as our data are in line with results obtained from *in vivo* recordings^12^ showing that SPW-R activity is affected after passing the high-mortality period around P24 in *Scn1a*^*+/−*^ mice.

### What are the cellular correlates of the ripple oscillation deficits?

PV-INs contribute to ripple oscillations and show prominent high-frequency AP activity both *in vivo* and *in vitro*.^20^^,^^33^^,^^34^^,^^47^ We found that the differences in ripple oscillation frequencies described by us and others^12^ were accompanied by changes in the SPW-R-induced AP firing of PV-INs within the same hippocampal slice preparation. This approach both improves consistency of our data and enables us to quantify APs directly evoked by SPW-R without possible alterations by current injection through the recording pipette. While the known coupling between APs of PV-INs and ripple oscillations seemed generally intact in all groups, recordings from young adult *Scn1a*^+/−^ mice showed an increased number of APs per SPW, likely due to the increased excitatory drive (Figure S3) received by CA3. The stronger excitation and higher number of APs in PV-INs are at the same time also expected to strengthen synaptic inhibition, which should adjust the excitation/inhibition balance and rather lead to higher ripple frequencies. The lower ripple oscillation frequencies observed in slices from *Scn1a*^*+/−*^ mice may thus be due to an altered synchronization of reciprocal inhibition in the PV-IN network.^20^ This may be further supported by the slightly lower consistency of AP (Figure 3) and IPSC (Figures 2 and 4) to ripple coupling suggested by the event cross-correlation analysis, which was not found for juvenile mice. Further experiments, such as simultaneous recordings from multiple cells during SPW-R, may be needed to address the cell-to-cell synchronization in detail.

The analysis of AP waveforms further confirmed the age-dependence of these deficits. For juvenile mice, it revealed moderate AP deficits during SPW-R, indicated by a slightly lower amplitude (by ∼5 mV) and a lower AP rising slope. As the differences were subtle but consistent, we think that APs of PV-INs start to be affected by the *Scn1a*-haploinsufficiency already at juvenile age (P19-23), which is compatible with previous reports showing Na_V_1.1 expression as early as P14^7^ and well before the described appearance of clearly Na_V_1.1-immunolabelled interneurons at ∼4 weeks of age.^48^

Data from surviving young adult mice showed stronger changes in AP waveforms such as amplitude and width, which affect the temporal dynamics of synaptic release and the resulting synchronization among PV-INs. Of note, both WT and *Scn1a*^+/−^ mice showed increased AP amplitudes and slopes compared to the juvenile groups, while the increase was lower for *Scn1a*^+/−^ mice. This is indicative of a reduced *Scn1a* upregulation in *Scn1a*^+/−^ mice,^48^ which may cause the more pronounced differences between young adult *Scn1a*^+/−^ and WT mice.

### Axon-specific AP waveform deficits

A reduction in the number of Na_V_ channels is expected to impair APs at the site of generation within the AIS. Somatic recordings represent a mixture of axonal components and backpropagating APs activating somatic Na_V_ channels. The observed inconsistency between somatic and axonal AP waveforms for juvenile mice may suggest that Na_V_1.1 upregulation in CA1 PV-INs^48^ may start in the soma and will only later accumulate in the AIS, where it provides a larger contribution to the axonal AP in young adult mice. Our findings emphasize the limitations of somatic recordings when quantifying Na_V_ channel deficits, as AP waveform and propagation may be affected differently between axon and soma, based on the compartment-specific Na_V_ channel subunit composition and density.

### How may the changes in AP waveform affect ripple oscillation frequencies?

Ripple oscillations reflect the compound inhibitory currents evoked by synchronized firing in the local PV-IN network.^20^ AP signaling is affected by Na_V_ channel deficits in several ways: (1) temporal variability in the input-output conversion may be increased,^49^ (2) AP initiation in the axon may be delayed, (3) AP propagation may be slowed down, (4) propagation failures may be more frequent,^24^ and (5) APs at synapses may be broadened and thus produce larger and/or less synchronized IPSCs.^50^ This may explain the slightly reduced coupling of IPSCs in our recordings from CA1 pyramidal cells (Figure 2). One caveat in the interpretation of these results is the fact that IPSCs collectively generate the summated ripple oscillations in the field signal, as mentioned above.^20^ Thus, IPSCs should always correlate well with the ripple in each slice, while a lower temporal coupling in individual cells may accumulate into a broadened compound IPSC. Future experiments may rather aim to quantify the correlation between groups of cells and respective ripple oscillations by recording APs and IPSCs from multiple cells at once during SPW-R instead of only from individual cells. In summary, all the factors mentioned above may jointly contribute to reduced ripple oscillation frequencies, which rely on the fast (∼200 Hz) and synchronous AP generation and propagation in PV-INs during SPW-R.^33^^,^^47^

### Fast-spiking vs. AP waveform deficits in *Scn1a*^*+/−*^ mice during SPW-R

Unexpectedly, the reduced generation of high-frequency firing reported for fast-spiking GABAergic interneurons based on current-evoked firing in several different brain regions including hippocampal CA1^8^^,^^10^^,^^29^^,^^30^^,^^31^^,^^51^ was not present in recordings from PV-INs during ongoing SPW-R activity in CA1 of the ventral hippocampus (Figure S4). Only when recording in slices from conventional incubation and storage, we found differences in maximum firing frequency, which followed the previously suggested age-dependence (Figure S5). In addition to possible differences in phenotype severity between colonies, our special experimental conditions may generally increase PV-IN excitability, as they are optimized for the generation of SPW-R activity with incubation at high oxygen levels and elevated temperatures. Further, a low input resistance (Table S2) at a high level of activity might also reduce the occurrence of the previously described depolarization block upon strong current injections.

Despite the absence of a strong fast-spiking deficit in slices generating SPW-R, we conclude that the observed changes in AP waveform are well compatible with an age-dependent deficit in Na_V_ channel function because of *Scn1a* haploinsufficiency. Interestingly, data from Valassina et al.^29^ may also suggest an age-dependent effect on AP waveform, as the AP rising and decay slope in their data are consistently reduced in young adults, but not in juvenile *Scn1a*^*+/−*^ mice, despite the fact that juvenile mice did show the fast-spiking deficit and young adults did not. Based on these data together with our own recordings, we conclude that mild reductions in AP rising slope and amplitude do not necessarily have a strong impact on the average AP frequency during repetitive firing in fast-spiking PV-INs. This may be due to the fact that a reduced rate of rise of single APs is influenced by the initial sodium channel pool, while the firing rate during repetitive firing mostly depends on the speed of recovery from inactivation. These features can thus be differentially modulated by changes in the size and subunit composition of the Na_V_ channel pool.

Our data rather indicate that the differences in ripple oscillations are caused by reductions in AP size and rising slope with increasing age as well as an increased AP duration instead of strong deficits in AP generation per se. The stability of AP generation, despite the apparent reduction in the availability of Na_V_ channels, is consistent with the high fidelity of firing in PV-INs and the high expression levels of axonal Na_V_ channels.^39^ It seems that the difference in fast-spiking is mainly due to strong depletion of the axonal Na_V_ channel pool at high frequencies, which may have limited consequences for network activity in the hippocampus (*in vivo*), at least for surviving mice with milder symptoms. In line with this hypothesis, De Stasi et al.^52^ reported normal firing activity of interneurons during spontaneous cortical activity *in vivo* despite the presence of the input-output deficit in their mice using their *in vitro* slice preparations. Further, a recent study by Capitano et al.^53^ describes hyperactivity rather than hypoactivity in both patients with DS and adult DS mice in the preictal phase associated with changes in network synchrony, which also has been reported previously.^54^ As our slice preparation also showed hyperactivity of PV-INs during SPW-R in our slice preparation for young adult *Scn1a*^*+/−*^ mice, it may thus seem a valuable model to further investigate these discrepancies between *in vitro* and *in vivo* data.

### Limitations of the study

Compensatory changes in ion channel expression and distribution could also contribute to the changes in network activity observed in *Scn1a*^*+/−*^ mice. It has previously been proposed that other Na_V_ subunits may be upregulated in response to partial, but permanent deletions of the Na_V_1.1 subunit.^8^ Further, neurons can homeostatically adjust their excitability by changing their expression level of postsynaptic receptors, ion transporters, and ion channels.^55^^,^^56^ Network activity is modulated by a distribution of variable synaptic strength so that changes in firing behavior in response to current injection seem insufficient to predict the recruitment into complex network oscillations. Changes of synaptic strength, as suggested by the larger trains of EPSCs received by PV-INs during SPW-R in young adult *Scn1a*^*+/−*^ may also contribute to changes in network synchronization and balance. This increase may be due to a higher activity of presynaptic CA3 pyramidal cells, possibly due to impaired inhibition in CA3 networks. Although we did not observe any correlation between SPW amplitude and ripple frequency within the given range, a stronger activation of PV-INs should, in principle, lead to stronger inhibition among PV-INs and onto pyramidal cells and not necessarily lower ripple oscillation frequencies. These changes in network activity may thus appear when activation of the network is not matched by adequate timing and strength of inhibition, which is the widely accepted concept regarding network dysfunctions in epilepsy.

## Resource availability

### Lead contact

Further information and requests for resources and reagents should be directed to and will be fulfilled by the lead contact, Fabian C. Roth (fabian.roth@medisin.uio.no).

### Materials availability

This study did not generate new unique reagents.

### Data and code availability

• Experimental data are publicly available on the EBRAINS data repository under https://doi.org/10.25493/YEDC-A5S.
• This paper does not report original code.
• All remaining data and any additional information required to reanalyze the data reported in this paper are available from the lead contact upon request.

## STAR★ Methods

### Key resources table

**Table:** 

REAGENT or RESOURCE	SOURCE	IDENTIFIER
**Chemicals, peptides, and recombinant proteins**		

Alexa Fluor 488 Hydrazide	Thermo Fisher/Invitrogen	Cat#A10436
DL-AP5	Bio-Techne/Tocris	Cat#0105
DNQX	Bio-Techne/Tocris	Cat#0189
SR95531 hydrobromide (gabazine)	Bio-Techne/Tocris	Cat#1262
Tetrodotoxin citrate (TTX)	Alomone Labs	Cat#T-550

**Deposited data**		

Experimental data underlying main and supplementary figures.	EBRAINS	https://doi.org/10.25493/YEDC-A5S

**Experimental models: Organisms/strains**		

Mouse: B6.129P2-Pvalb^tm1(cre)Arbr^/J	Jackson Laboratory	RRID:IMSR_JAX:017320
Mouse: B6.Cg-Gt(ROSA)26Sor^tm14(CAG-tdTomato)/Hze^/J	Jackson Laboratory	RRID:IMSR_JAX:007914
Mouse: 129S-Scn1a^tm1Kea^/Mmjax	MMRRC	RRID:MMRRC_037107-JAX

**Software and algorithms**		

MATLAB	The Mathworks, Inc	https://www.mathworks.com/; RRID: SCR_001622
GraphPad Prism 10	GraphPad Software	http://www.graphpad.com; RRID:SCR_002798
Inkscape		https://inkscape.org/; RRID:SCR_014479
pClamp 9.2.1.9	Molecular Devices	RRID:SCR_011323

**Other**		

Multiclamp 700B Amplifier	Molecular Devices	RRID:SCR_018455
EXT-10F field potential amplifier	NPI electronic	https://www.npielectronic.com/product/ext-10-2f/; RRID:SCR_016002

### Experimental model and study participant details

#### Animals

All experimental procedures were approved by the Norwegian Food Safety Authority (FOTS project number 19130, 30123) and were in accordance with national laws and the EU Directive 2010/63 for the protection of animals used for scientific purposes.

*Scn1a* heterozygous knockout mice and WT littermates (129S-Scn1a^tm1Kea^/Mmjax, MMRRC strain #037107-JAX) were purchased from MMRRC as a rederived litter (The Jackson Laboratory, Bar Harbor, ME. USA). A colony was maintained by crossing heterozygous 129S.*Scn1a*^*+/−*^ with WT 129S.*Scn1a*^*+/+*^ mice.^8^^,^^30^ For investigation of the *Scn1a* haploinsufficiency in combination with transgenic labeling of PV-expressing cells, male heterozygous 129S.*Scn1a*^*+/−*^ were crossed with female B6.PV-Cre.tdTomato mice. Double homozygous transgenic B6.PV-Cre.tdTomato mice were obtained by crossing B6-PVcre (B6.129P2-Pvalb^tm1(cre)Arbr^/J, JAX strain #017320) with Ai14 reporter mice (B6.Cg-Gt(ROSA)26Sor^tm14(CAG-tdTomato)Hze^/J, JAX strain #007914) for 2 generations (Figure S1).

This breeding scheme provided, on average, an equal number of offspring carrying either one (*Scn1a*^+/−^) or two copies (*Scn1a*^+/+^, referred to as WT) of the *Scn1a* gene, while all mice carried one copy each of cre-recombinase and Rosa.tdTomato. All offspring of either sex was used for experiments for comparison between *Scn1a*^+/−^ and WT mice (*Scn1a*^+/−^, 45% female and 55% male mice; WT, 50% female and 50% male mice). We did not detect sex-specific differences in our dataset. Mice were used at ages between postnatal day (P) 19–23 (juvenile mice) and ages between P28-37 (young adult mice, “survivors”), as indicated in each figure legend and table. *Scn1a*^+/−^ mice showed a ∼21% mortality when housed beyond P24, comparable to mortalities described for this Dravet Syndrome mouse model.^8^^,^^24^^,^^57^

Mice were weaned at P21 and housed with their littermates of the same sex. Water and food were available *ad libitum*, and mice were housed on a 12 h light 12 h dark cycle (lights on at 7 a.m).

The genotype of all mice was determined by PCR using ear biopsies obtained at ages between P15-28. In some cases, the genotype was determined after the mice had been sacrificed for slice preparation by using tail snips. There were no genotyping errors.

### Method details

#### Preparation of mouse brain slices

Mice were anesthetized with isoflurane and decapitated. Brains were quickly removed and transferred into ice-cold sucrose-containing cutting solution (for solution recipe see below). After trimming, tissue blocks containing the hippocampus were glued upon the dorsal face onto a platform. Horizontal slices of the ventral and intermediate hippocampus were cut at 400 μm thickness using a vibratome (VT1200S, Leica Microsystems) and incubated at ∼34°C for at least 2 h in a custom-built Haas-type interface storage chamber^58^ perfused with standard physiological extracellular solution. The inside of the chamber around the perfused slices was saturated with humidified carbogen (5% CO_2_ in O_2_). The incubation in the interface chamber led to stable SPW-R network activity,^27^ as well as an improved survival and quality of superficial cells in slices from juvenile and young adult mice.

For recordings in slices without SPW-R activity, we cut horizontal slices of the ventral to intermediate hippocampus at 350 μm thickness and incubated them on nylon nets within a conventional beaker filled with oxygenated recording solution at 32°C for 30 min, followed by storage at room temperature.

#### Field recordings

For electrophysiological recordings, slices were transferred to a custom-built double perfusion chamber^26^ mounted in an upright microscope (Olympus BX51WI) and perfused with standard recording solution at a speed of 4 mL/min. The temperature of the bath solution was adjusted to ∼32°C using an inline-heater, and the recording temperature of was continuously monitored with a micro thermistor positioned in the vicinity of the slice (both Sigmann Elektronik, Hueffenhardt, Germany).

Extracellular field potentials were registered using ACSF-filled borosilicate glass electrodes (tip diameter 3–5 μm) inserted ∼100 μm deep into the somatic layer. Signals were amplified 1000x using a Multiclamp 700B amplifier (Molecular Devices, San Jose, USA) or an EXT-10F amplifier (npi electronic, Tamm, Germany), low-pass filtered at 2–8 kHz, high-pass filtered at 0.1 Hz, digitized at 20 kHz with an analog-to-digital converter (ADC, Digidata 1322 converter board, Molecular Devices), and saved on a computer using pClamp software.

#### Patch-clamp recordings

PV-INs in CA1 stratum pyramidale were identified based on tdTomato fluorescence, and all cells identified by tdTomato were fast-spiking. Current- and voltage-clamp recordings were performed using a Multiclamp 700B amplifier (Molecular Devices). Pipette capacitance and series resistance compensation (bridge balance) were applied throughout current-clamp experiments. The bridge balance was between 10 and 25 MΩ, while no correlation was found between bridge balance and AP amplitude values and there was no difference in bridge balance values between groups. A holding current was injected at the soma (range 0 to −250 pA) of PV-INs to stabilize the membrane potential at approximately −65 mV. IPSCs were recorded from PV-INs and CA1 pyramidal cells in voltage clamp mode with a holding potential of 0 mV, EPSCs at a holding potential of −65 mV.

Signals were low-pass filtered at 10 kHz in current clamp and at 4 kHz in voltage-clamp recordings, sampled at 20–100 kHz using a digitizer (Digidata 1322 converter board, Molecular Devices). Pulse protocols were generated using the pClamp software (Molecular devices). All voltage- and current-clamp recordings were performed at near-physiological temperature (range: 31°C–33°C). Patch pipettes were produced from thick-walled borosilicate glass capillaries (outer diameter = 2 mm, inner diameter = 1 mm) with a horizontal pipette puller (P-97, Sutter Instruments). When filled with internal solution, pipettes had a resistance of 4–6 MΩ for somatic recordings and 10–16 MΩ for axonal recordings.

#### SPW-R-independent patch-clamp recordings

To record input-output properties of PV-INs and spontaneous synaptic inhibition to CA1 pyramidal cells independently of SPW-R activity, we used beaker-incubated slices in a conventional recording chamber without double perfusion at 32°C with a perfusion speed of 2.5 mL/min. Spontaneous IPSCs (sIPSCs) were recorded in voltage clamp mode with a holding potential of −60 mV in the presence of synaptic blockers for AMPA (20 μM DNQX) and NMDA (20 μM AP-V) glutamate receptors. For recording miniature IPSCs (mIPSCs), we used the same approach with the addition of 1 μM TTX. To verify the identity of IPSCs, we also blocked GABA_A_ receptors with 4 μM SR95531 hydrobromide (gabazine) at the end of one recording.

#### Dual soma-axon recordings

For axonal recordings from interneurons, we applied the following experimental strategy. First, a PV-IN with a suitable proximal axon bleb at very short distance from the soma (typically 20–50 μm) was selected based on the visual appearance of axon and cell body in the tdTomato and the IR-DIC image. The respective soma was then filled with Alexa Fluor 488 hydrazide (Invitrogen, Thermo Fisher Scientific, Oslo, Norway) using the whole-cell patch clamp configuration. Second, after 5 min, the proximal axon was traced using the green fluorescence signal to verify that the previously identified axon was connected to the soma. Unmyelinated cut-ends of the axon (“blebs”) were patched under IR-DIC contrast-enhanced illumination with a second small pipette optimized for subcellular recordings. During the recordings, the time of light exposure was minimized to avoid phototoxic damage. Dual recordings of somata and axons were used to record voltage responses and AP firing in response to step current injections in current clamp mode delivered to either the soma or the axon.^25^

#### Solutions

The cutting solution contained: 87 mM NaCl, 25 mM NaHCO3, 25 mM glucose, 75 mM sucrose, 2.5 mM KCl, 1.25 mM NaH_2_PO_4_, 0.2 mM CaCl_2_ and 3 mM MgCl_2_ (equilibrated with 95% O_2_/5% CO_2_ gas mixture).

The extracellular solution (artificial cerebrospinal fluid, ACSF) for storage and recording of slices contained: 125 mM NaCl, 25 mM NaHCO_3_, 25 mM glucose, 2.5 mM KCl, 1.25 mM NaH_2_PO_4_, 2 mM CaCl_2_ and 1 mM MgCl_2_ (equilibrated with 95% O_2_/5% CO_2_ gas mixture).

The standard intracellular solution for patch-clamp experiments contained: 120 mM potassium D-gluconate, 20 mM KCL, 10 mM EGTA, 10 mM HEPES, 2 mM MgCl_2_, 2 mM Na_2_ATP, 0.3 mM NaGTP, 3 mM K-phosphocreatine. For recordings of sIPSCs and mIPSCs, the intracellular solution contained: 130 mM CsCl, 0.5 mM CaCl2, 10 mM HEPES, 10 mM EGTA, 4 mM MgATP, 0.5 mM NaGTP, 5 mM Na_2_-phosphocreatine, 2 mM QX-314.

#### Chemicals

For a partial block (∼50%) of Na_V_ channels in a subset of current-clamp experiments,^39^^,^^40^ 4 nM Tetrodotoxin Citrate (TTX; Alomone Labs, Jerusalem, Israel) was added to the recording solution. To record spontaneous IPSCs in pyramidal cells, 20 μM AP-V and 20 μM of DNQX (both Bio-Techne/Tocris) was added to the recording solution and for recordings of miniature IPSC, 1 μM TTX was added in addition. To confirm the identity of IPSCs, 4 μM SR95531 hydrobromide (gabazine, Bio-Techne/Tocris) was added in addition at the end of one experiment.

### Quantification and statistical analysis

#### Analysis of electrophysiological data

Data were analyzed using custom-made routines in MATLAB (The Mathworks, Natick, MA). SPW-R were detected from low-pass filtered (60 Hz) field potential raw data by finding local maxima above 0.05 mV within 30 ms time windows. This threshold exceeds baseline noise by about four standard deviations, yielding reliable detection of SPW-R, as confirmed by visual inspection of traces. High-frequency ripple oscillations were analyzed with continuous wavelet transform (complex Morlet wavelet), starting 33 ms before and ending 67 ms after the peak of each SPW-R.^19^ The resulting wavelet spectrogram was used to quantify the leading ripple frequency (the most prominent frequency above 140 Hz). The number of ripple cycles, represented by the number of ripple troughs that occur during an SPW-R event, was detected by band-pass filtering at 150–300 Hz.

The temporal relationship between APs and ripple cycles was analyzed by calculating event-based cross correlations between detected APs in the whole cell recordings and individual ripple troughs within each SPW-R.^23^ Each delay value in positive or negative direction was added to a correlation histogram for each set of field and whole cell recordings. Multiple peaks form in the histogram when multiple counts of similar delays accumulate for APs preceding or following ripple cycles, suggesting rhythmic comodulation of the two sets of events. For quantification of the degree of temporal coupling and the coupling precision, the central peak closest to 0 ms delay was quantified by (1) its maximum bin count relative to the total number in percent and (2) its width in milliseconds by measuring the distance between the 25^th^ and 75^th^ percentile of a cumulative distribution of values within the central peak (lower values correspond to more precise coupling).^23^ For group comparisons, we normalized the AP number per SPW-R by dividing the median AP number per SPW-R by the respective median SPW amplitude for each cell to account for the known correlation between SPW amplitude and the strength of synaptic input.^27^

The same analysis was used to assess the temporal coupling between individual ripple troughs and IPSCs recorded from CA1 pyramidal cells. For the event cross correlation analysis, IPSC events were automatically detected by deconvolution of the raw signal with a Wiener filter (deconvwnr, MATLAB). A single kernel was calculated from a double exponential peak function fitted to IPSC kinetics.^27^^,^^59^ For quantification of IPSC magnitude, the charge was quantified as the integral of each IPSC train as “area under the curve” in coulomb (A×s). For group comparisons, we then normalized the data by dividing median IPSC charge values by the respective median SPW amplitudes for each cell to account for the linear correlation between SPW amplitude and IPSC charge.^27^

For the analysis of input-output relationships, only APs occurring within the stimulus pulse were considered, excluding those arising from spontaneous neuronal activity. APs were identified and analyzed using custom-made scripts (MATLAB). APs with an amplitude of less than 20 mV were excluded from further analysis. AP threshold was defined as the first data point above a rising slope of 50 V/s, while the amplitude was quantified as the difference between the threshold and the peak of the AP. The width of each AP was determined by calculating the time difference between (1) the first point before the half-maximum amplitude on the ascending phase and (2) the subsequent data point below this value on the descending phase. For SPW-R-evoked APs, only the first AP was included in the analysis. For current-evoked APs, the first AP of a train of at least 250 Hz was analyzed except for experiments with TTX application where the 5^th^ AP in a train of 100–130 Hz was analyzed for group comparisons.

#### Statistical analysis

Statistical analysis was performed using MATLAB or Prism 10 (GraphPad, Boston, USA). Values in text are presented as mean ± standard error of the mean (SEM). Sample sizes (n numbers) generally indicate number of mice, unless stated otherwise in the figure legend. Normality of the data was tested using the Shapiro-Wilk test (Prism 10). Given normal distribution, Welch’s *t* test was used to define statistical significance. Otherwise, the Mann-Whitney U test was used where indicated. Input-output relationships of AP firing were compared using ANOVA with Sidak’s multiple comparisons test. Significant differences are presented as follows: ∗ = *p* < 0.05, ∗∗ = *p* < 0.01, ∗∗∗ = *p* < 0.001. *p* values are reported in the text.

To avoid bias, experimenters were blind to animal genotype during preparation of slices and recordings.

## Acknowledgments

This work was supported by the 10.13039/501100005416Research Council of Norway (grant no. 315935) to R.L. and F.C.R. We thank Dr. Ethan M. Goldberg for help with establishing the *Scn1a*^*+/−*^ mouse colony. We also thank Drs. Hua Hu, Andreas Draguhn and Sami Hassan for critically reading a previous version of the manuscript, as well as Cecilie Petterson Oksvold and Gaute Nesse for genotyping of mice. We thank the animal facility (KPM-UiO) for mouse colony management.

## Author contributions

R.L. investigation, data curation, formal analysis, visualization, writing—original draft, and writing—review and editing. F.C.R. conceptualization, methodology, funding acquisition, supervision, project administration, investigation, data curation, formal analysis, visualization, writing—original draft, and writing—review and editing.

## Declaration of interests

The authors declare no competing interests.
