# Supplementary material for: Age-dependent axonal dysfunctions and altered sharp-wave ripple oscillations in Scn1a+/− mice
Source: iScience. 2026 Jul 21;29(8):116784. doi: 10.1016/j.isci.2026.116784 (PMC13392871; doi:10.1016/j.isci.2026.116784)
Supplement: Document S1. Figures S1–S6 and Tables S1, and S2 [file mmc1.pdf]

iScience, Volume *29*

Supplemental information

Age-dependent axonal dysfunctions and altered sharp-wave ripple oscillations in *Scn1a*$^{+/-}$ mice

Raquel Lascorz and Fabian C. Roth

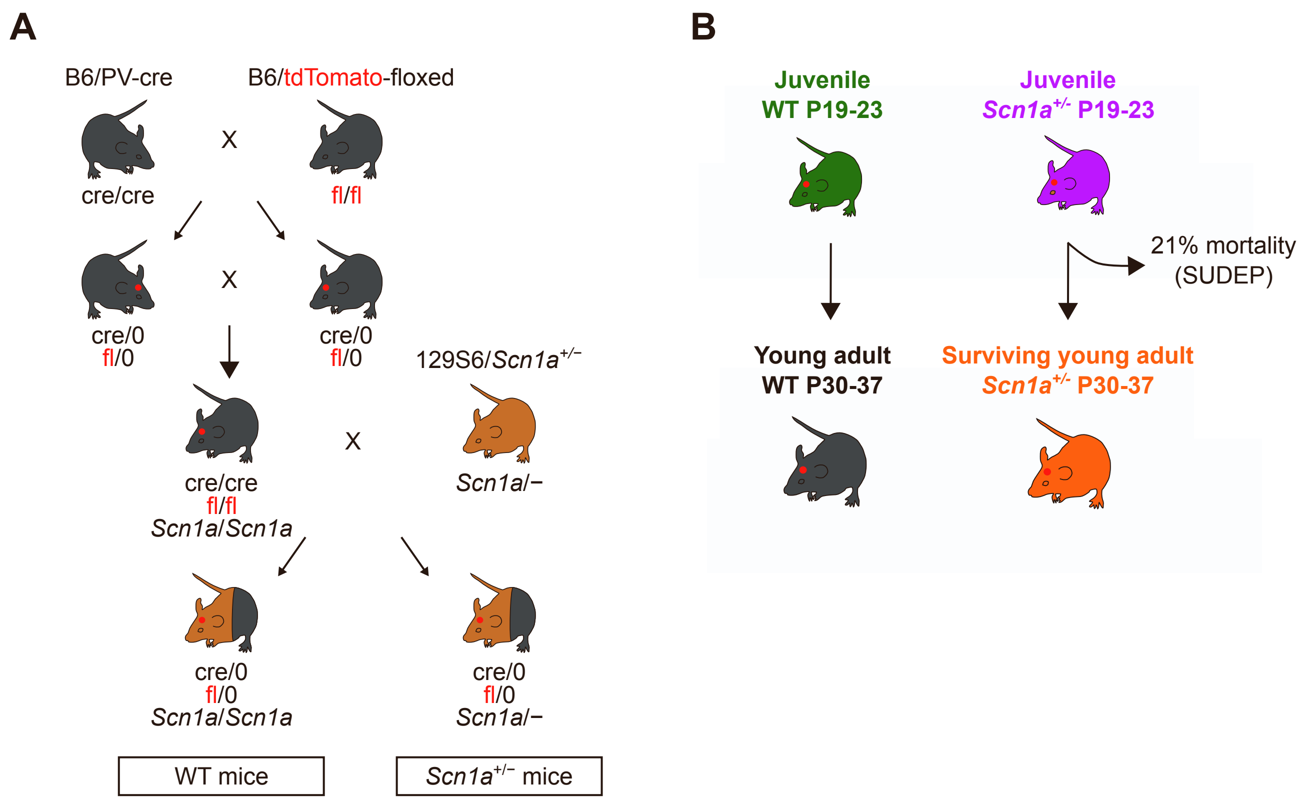

Supplementary Figure 1. Experimental approach for transgenic mice.
(**A**) Breeding scheme for the generation of triple transgenic mice. A colony of homozygous mice expressing tdTomato in parvalbumin (PV)-expressing cells was generated by crossbreeding and backcrossing C57BL/6 PV-cre (B6.129P2-Pvalb$^{tm1(cre)Arbr}$/J) mice with Ai14 (B6.Cg-Gt(ROSA)26Sor$^{tm14(CAG\text{-}tdTomato)Hze}$/J) mice for two generations. The double homozygous PV-cre.tdTomato-flox C57BL/6 mice were then crossbred with 129S6/Scn1a$^{+/-}$ (129S-Scn1a^{tm1Kea}/Mmjax) mice to generate, on average, an equal amount of WT and Scn1a$^{+/-}$ mice. All mice resulting from this breeding have a 50% C57BL/6 and 50% 129S6 genetic background and express tdTomato in PV-expressing cells.
(**B**) Illustration of experimental groups. Offspring from the last breeding step in (A) was used for experiments in two different age groups. WT and *Scn1a*$^{+/-}$ mice were used for recordings at ages of 19-23 days. Because of the mortality (sudden unexpected death in epilepsy, SUDEP) of *Scn1a*$^{+/-}$ mice on the mixed BL6/129S6 genetic background around 24 days of age, only surviving *Scn1a*$^{+/-}$ mice could be used for recordings at ages of 30-37 days. Color coding matches respective traces and summary plots throughout all figures.

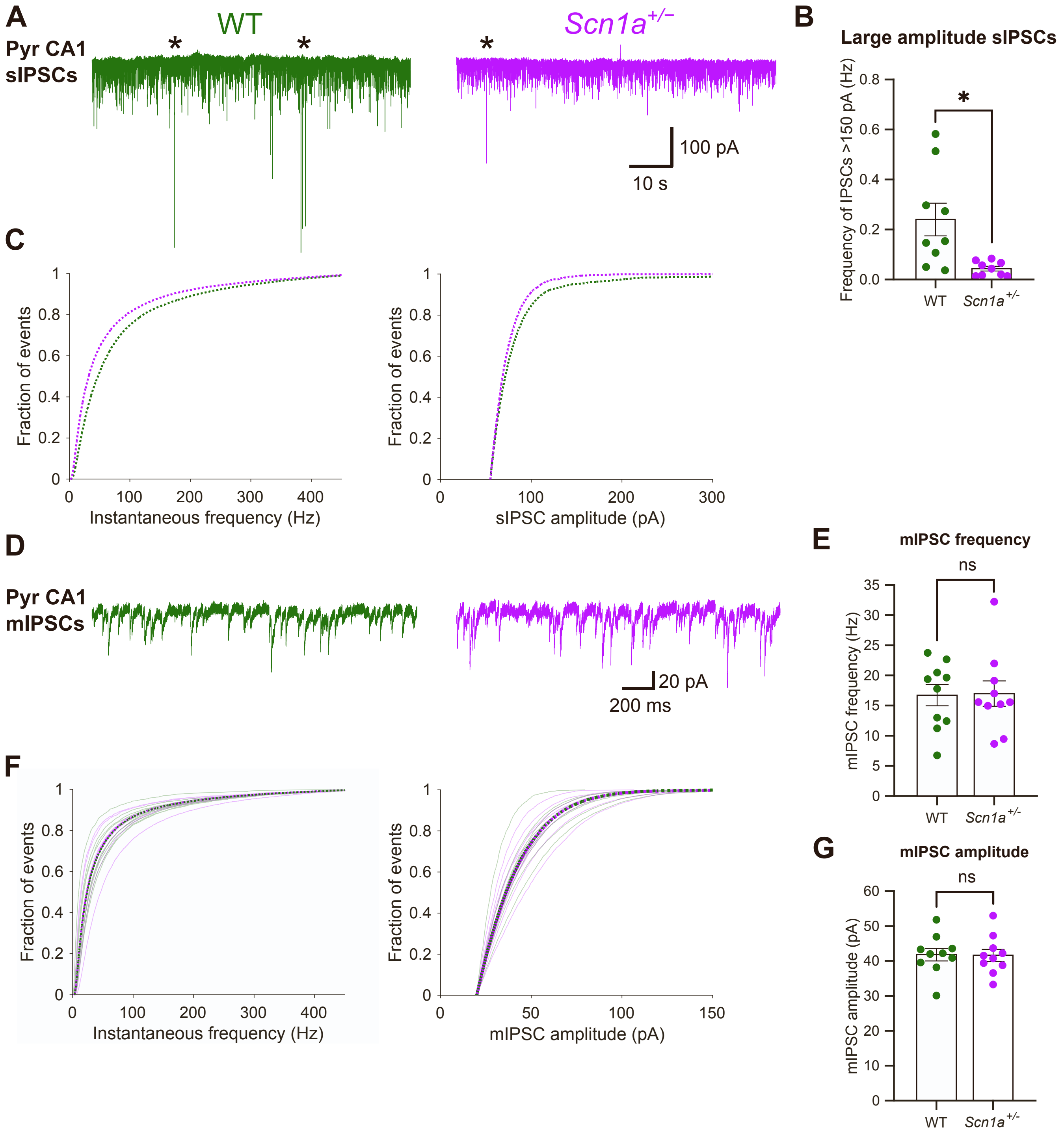

Supplementary Figure 2. Without SPW-R activity, CA1 pyramidal cells from *Scn1a*$^{+/-}$ mice receive fewer spontaneous IPSCs, but equal levels of AP-independent miniature IPSCs, related to Fig. 2.
(**A**) Voltage clamp recordings (-60 mV holding potential) of spontaneous IPSCs (sIPSCs) in CA1 pyramidal cells from WT (green) and *Scn1a*$^{+/-}$ mice (magenta). Presumably AP-induced large IPSCs are indicated with asterisks.
(**B**) Summary plot for the frequency of large-amplitude sIPSCs (>150 pA in amplitude).
(**C**) Mean cumulative distribution for overall sIPSC frequencies (left) and for sIPSC amplitudes (>60 pA, right).
(**D**) Similar to (A), but in 1 µM TTX for recording miniature IPSCs (mIPSCs).
(**E**) Summary plot for the frequency of mIPSCs.
(**F**) Cumulative distributions for mIPSC frequencies (left) and amplitudes (right), thick dotted lines indicate the respective mean distributions.
(**G**) Summary plot for mIPSC amplitudes.
For (B,C), summary data from n=9 cells from 6 WT mice (green) and n=9 cells from 5 *Scn1a*$^{+/-}$ mice (magenta) aged P21-23.
For (E-G), summary data from n=10 cells from 6 WT mice (green) and n=10 cells from 5 *Scn1a*$^{+/-}$ mice (magenta) aged P21-23.
ns = p>0.05, * = p<0.05, Welch's t test. Data are represented as mean ± SEM.

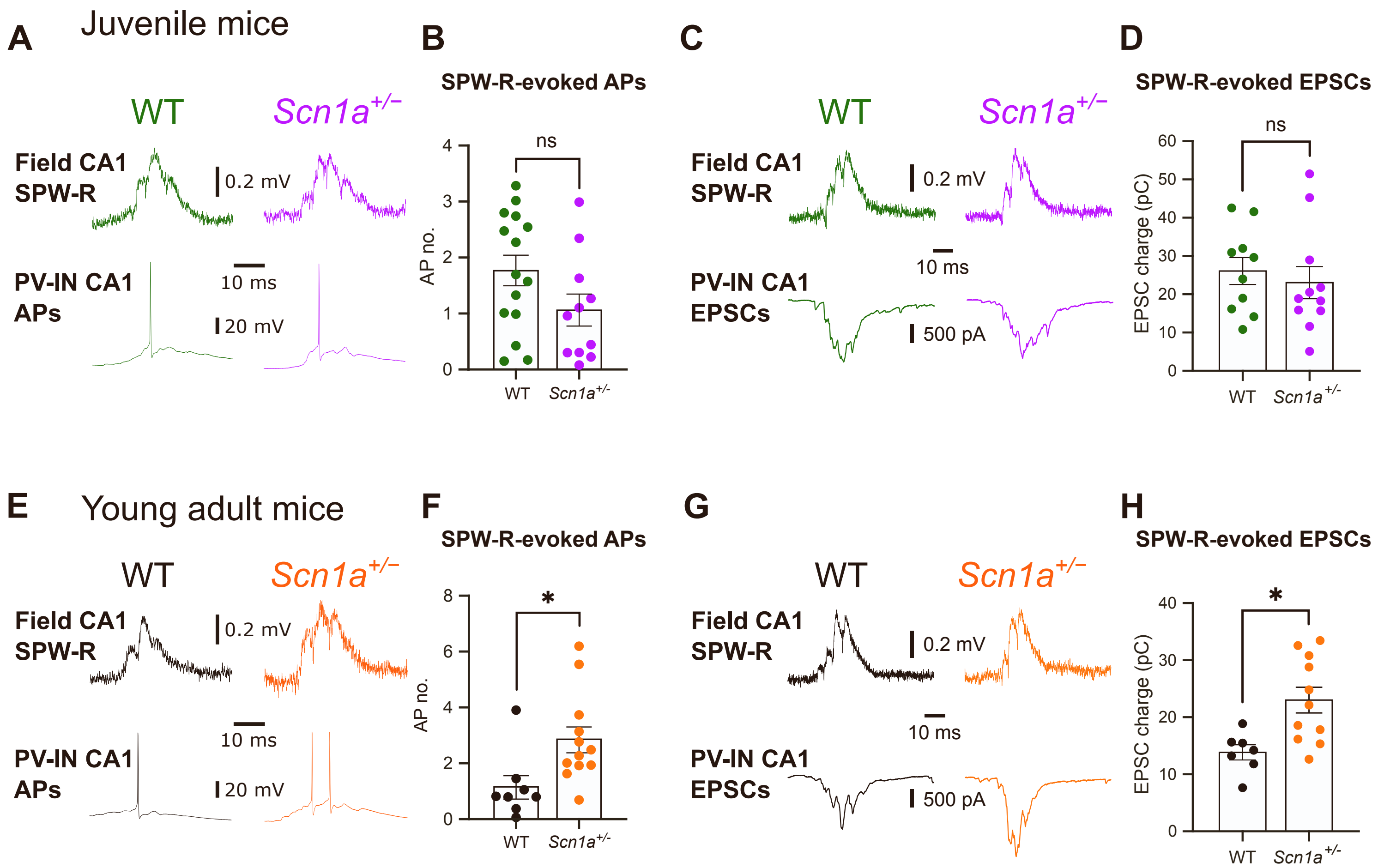

Supplementary Figure 3. SPW-R-evoked AP firing and EPSC charge in CA1 PV-INs are increased in young adult *Scn1a*$^{+/-}$ mice, related to Fig. 3.
(**A**) Representative traces of field (upper traces) and whole cell current clamp recordings (lower traces) from slices from juvenile WT (green) and *Scn1a*$^{+/-}$ (magenta) mice showing SPW-R-evoked APs.
(**B**) Summary data for the mean number of APs per SPW-R for both genotype groups in juvenile mice.
(**C**) Representative traces of field (upper traces) and whole cell voltage clamp (-65 mV holding potential) recordings (lower traces) from slices from juvenile WT (green) and *Scn1a*$^{+/-}$ (magenta) mice showing SPW-R-associated EPSC trains.
(**D**) Summary data for the mean EPSC charge per SPW-R for both genotype groups in juvenile mice.
(**E-H**) Similar to (A-D), but for data from young adult WT (black) and *Scn1a*$^{+/-}$ (orange) mice.
For (B), summary data from n=15 WT and n=11 *Scn1a*$^{+/-}$ mice aged P19-22.
For (D), summary data from n=10 WT and n=11 *Scn1a*$^{+/-}$ mice aged P19-22.
For (F), summary data from n=8 WT and n=12 *Scn1a*$^{+/-}$ mice aged P30-37.
For (H), summary data from n=7 WT and n=11 *Scn1a*$^{+/-}$ mice aged P30-37.
ns = p>0.05, * = p<0.05, Welch's t test. Data are represented as mean ± SEM.

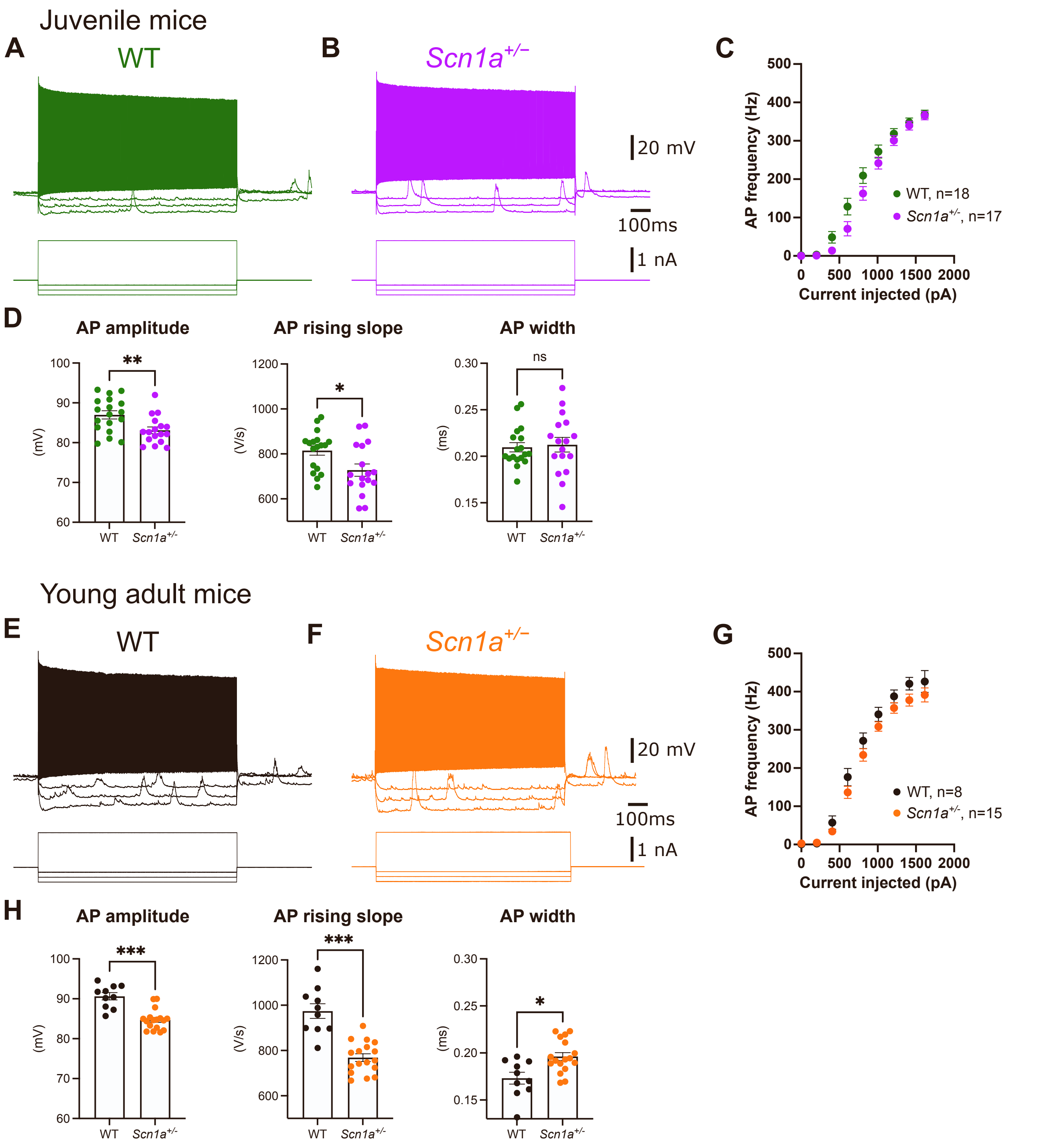

Supplementary Figure 4. Input-output relationships in PV-IN of WT and *Scn1a*$^{+/-}$ mice in slices generating SPW-R are similar despite differences in AP waveforms, related to Figs. 3 and 5.
(**A**) Representative traces of voltage responses to step current injections for a PV-IN from a juvenile WT mouse.
(**B**) Similar traces as in (A), but for a PV-IN from a juvenile *Scn1a*$^{+/-}$ mouse.
(**C**) Summary plot for the input-output relationship for WT mice (green) and *Scn1a*$^{+/-}$ mice (magenta).
(**D**) Summary plots for AP amplitude, AP rising slope, AP width, and AP decay slope for the first AP of a high frequency train for WT and *Scn1a*$^{+/-}$ mice.
(**E-H**) Similar to (A-D) showing representative traces for recordings obtained from young adult WT (black) and *Scn1a*$^{+/-}$ mice (orange) and respective summary plots.
For (C-D), summary data from n=18 WT mice, n=17 *Scn1a*$^{+/-}$ mice aged P19-22.
For (G-H), summary data from n=8 WT mice, n=15 *Scn1a*$^{+/-}$ mice aged P30-37.
ns = $p>0.05$, * = $p<0.05$, ** = $p<0.01$, *** = $p<0.001$, Welch's t test.
Data are represented as mean ± SEM.

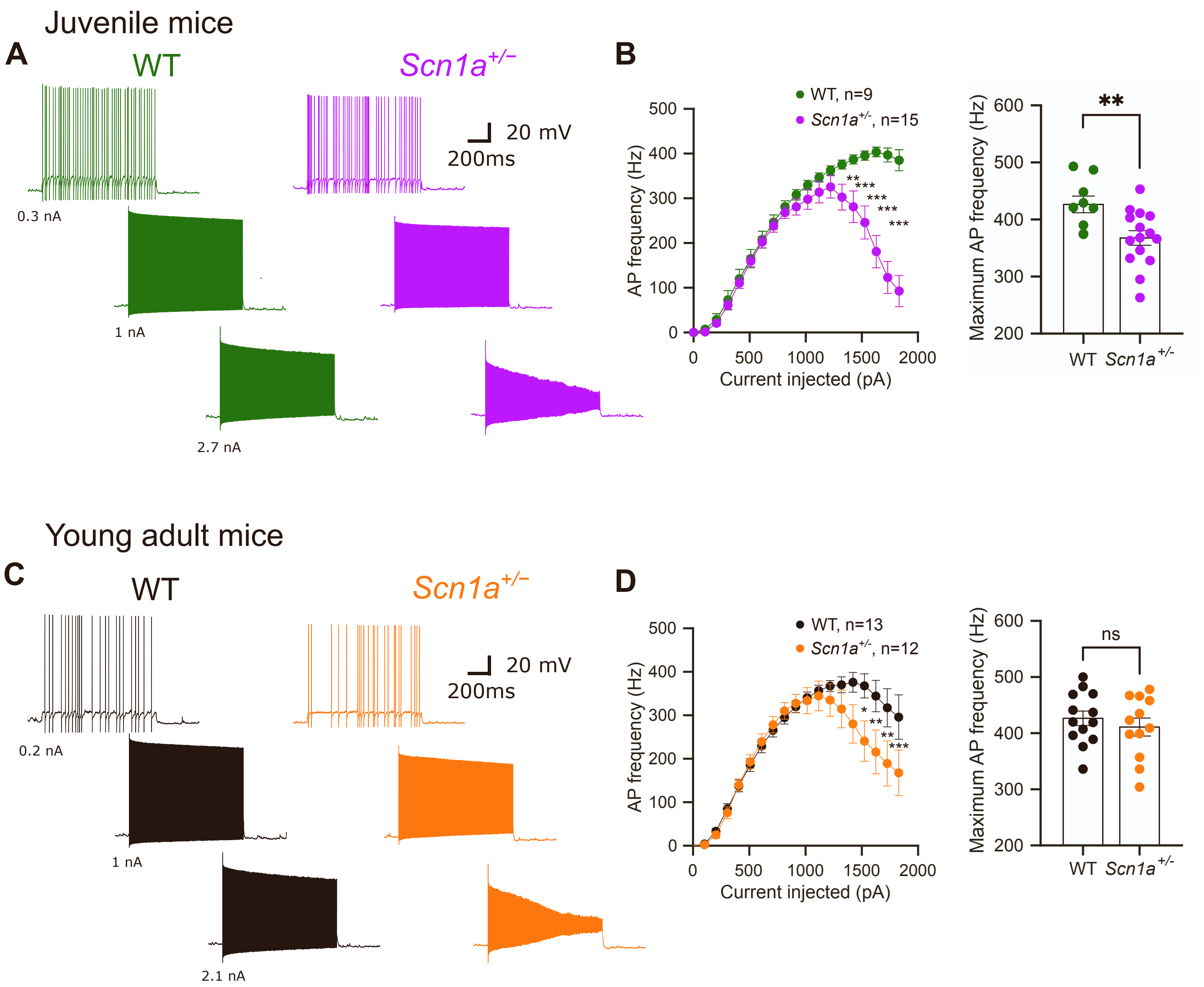

Supplementary Figure 5. Input-output relationships for CA1 PV-IN of juvenile *Scn1a*$^{+/-}$ mice in slices without SPW-R show reduced maximum firing, related to Fig. 3 and Suppl. Fig. 4.
(**A**) Example traces of voltage responses to three step current injections (0.3, 1, 2.7 nA) in PV-INs from juvenile WT (green) or *Scn1a*$^{+/-}$ (magenta) mice. Cells were chosen to have similar firing frequencies at the lowest (0.3-nA) pulse.
(**B**) Summary plot for the input-output relationship (left) and maximum AP firing frequency (right) in PV-IN from WT mice (green) and *Scn1a*$^{+/-}$ mice (magenta).
(**C**) Example traces of voltage responses to threestep current injections (0.2, 1, 2.1 nA) in PV-INs from young adult WT (black) or *Scn1a*$^{+/-}$ (orange) mice. Cells were chosen to have similar firing frequencies at the lowest (0.2-nA) pulse.
(**D**) Similar to B, but for data from young adult mice.
For (B), summary data from n=9 cells from 4 WT mice, n=15 cells from 5 *Scn1a*$^{+/-}$ mice aged P20-21.
For (D), summary data from n=13 cells from 5 WT mice, n=12 cells from 5 *Scn1a*$^{+/-}$ mice aged P30-32.
ns = p>0.05, * = p<0.05, ** = p<0.01, *** = p<0.001, ANOVA with Sidak's multiple comparisons test for IV-curves, Welch's t test for maximum AP frequency. Data are represented as mean ± SEM.

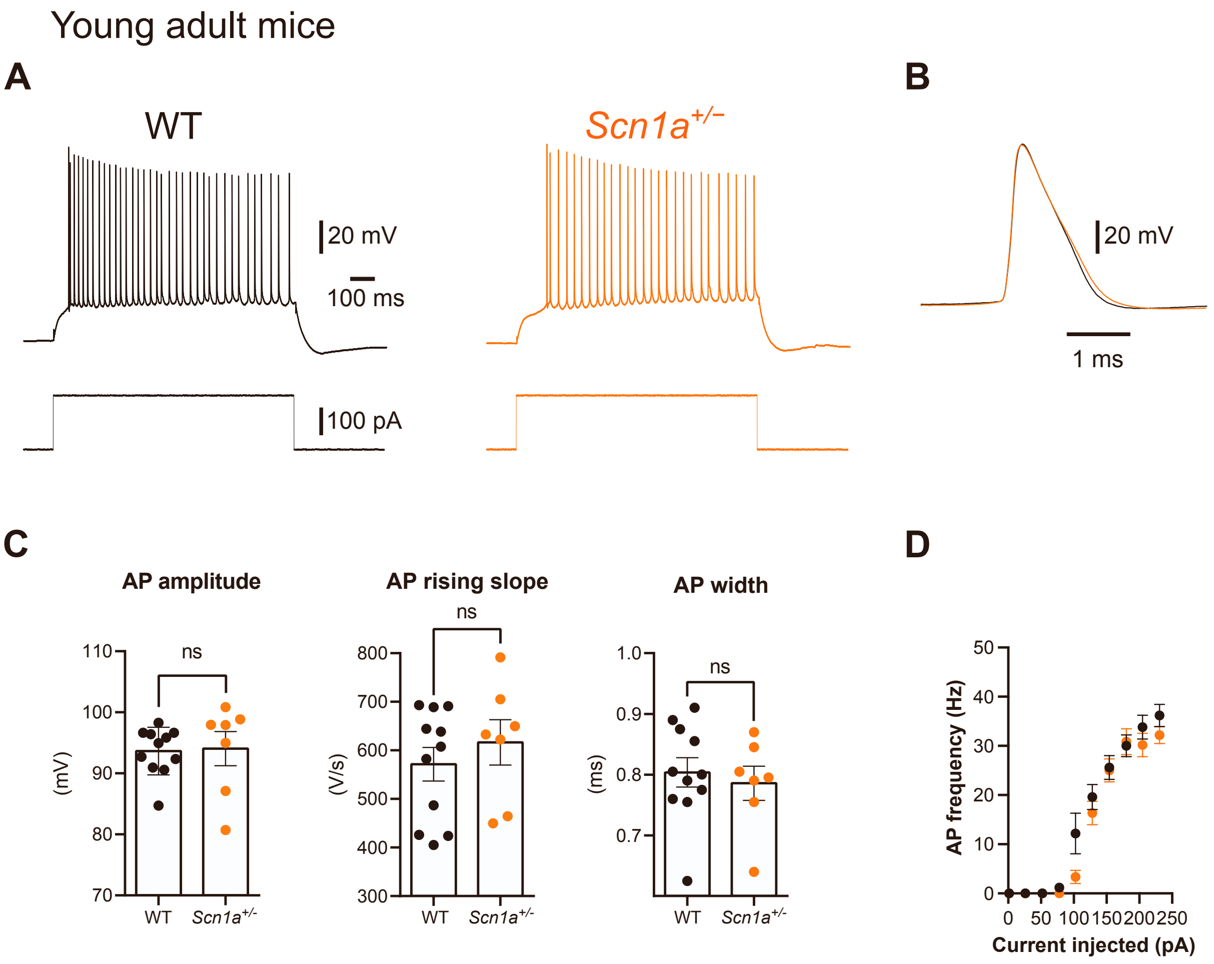

Supplementary Figure 6. No difference in action potential waveform between CA1 pyramidal cells from WT vs. *Scn1a*$^{+/-}$ mice, related to Fig. 2 and Suppl. Fig. 4.

(**A**) Representative somatic firing patterns of CA1 pyramidal cells recorded from young adult WT (left, black traces) and from *Scn1a*$^{+/-}$ mice (right, orange traces) in response to current injection shown below.

(**B**) Overlay of the first APs of respective traces shown in (A) at an expanded time scale.

(**C**) Summary plots for AP amplitude, AP rising slope, and AP width. Values are presented in the main text (WT mice n = 11; *Scn1a*$^{+/-}$ mice n=7, aged P28-37).

(**D**) F-I curve for a subset of pyramidal cells (WT mice n=5; *Scn1a*$^{+/-}$ mice n=6).

ns = p>0.05, Welch's t test. Data are represented as mean ± SEM.

Supplementary Table 1. Key parameters of SPW-R-induced APs in CA1 PV-INs (related to Fig. 5)

	Juvenile, P19-22			*Young adult,* P30-37		
	WT mice (n = 15)		*Scn1a*$^{+/-}$ mice (n = 11)	WT mice (n = 8)		*Scn1a*$^{+/-}$ mice (n = 12)
Resting membrane potential (mV)	-64.84 ± 0.31		-65.14 ± 0.54	-65.56 ± 0.28		-65.40 ± 0.42
AP threshold (mV)[1]	-39.38 ± 0.58		-40.52 ± 0.93	-40.17 ± 1.14		-39.06 ± 0.72
AP amplitude (mV)[2]	**86.60 ± 1.11**	*	**81.80 ± 1.33**	**90.70 ± 1.23**	**	**83.59 ± 1.05**
AP width (μs)[3]	206.97 ± 6.03		206.01 ± 6.63	**166.17 ± 5.75**	**	**191.36 ± 4.40**
Max AP rising slope (V/s)[4]	**755.77 ± 22.28**	*	**677.92 ± 29.17**	**913.41 ± 30.60**	**	**730.70 ± 20.26**
Max AP decay slope (V/s)[5]	494.37 ± 20.66		471.71 ± 22.08	**644.57 ± 23.32**	**	**531.50 ± 18.10**
AHP amplitude (mV)[6]	-19.57 ± 0.77		-17.83 ± 0.88	-22.00 ± 0.76		-19.98 ± 0.73

AP properties are represented as mean ± SEM of medians of parameters measured from the first AP occurring during a SPW-R. AP, action potential; AHP, afterhyperpolarization.

[1]Membrane potential at the point where AP rate of rise surpassed 50 V/s
[2]Voltage difference between AP threshold and peak
[3]Full width at half maximum amplitude
[4]Maximum rate of rise during the AP rising phase
[5]Maximum rate of decay during the AP decaying phase
[6]Peak negative voltage deflection after the AP relative to the threshold

* = $p<0.05$; ** = $p<0.01$

Supplementary Table 2. Current-induced electrical parameters of CA1 PV-INs (related to Supplementary Fig. 4)

	Juvenile, P19-22			*Young adult,* P30-37		
	WT mice (n = 18)		*Scn1a*$^{+/-}$ mice (n = 17)	WT mice (n = 10)		*Scn1a*$^{+/-}$ mice (n = 17)
Resting membrane potential (mV)	-65.28 ± 0.29		-65.62 ± 0.38	-64.50 ± 0.26		-64.91 ± 0.58
Input resistance (MΩ)	39.62 ± 3.54		32.30 ± 2.25	37.03 ± 3.42		33.17 ± 2.39
Capacitance (pC)	198.32 ± 17.28		185.53 ± 22.41	135.33 ± 17.17		126.24 ± 16.47
Latency to 1st AP (ms)	274.07 ± 74.27		357.19 ± 78.28	294.47 ± 83.64		347.44 ± 53.22
AP threshold (mV)[1]	-46.11 ± 0.69		-46.43 ± 0.58	-46.99 ± 1.12		-47.07 ± 0.54
AP amplitude (mV)[2]	**86.98 ± 1.02**	**	**83.11 ± 0.85**	**90.63 ± 0.91**	**	**84.71 ± 0.62**
AP width (µs)[3]	209.67 ± 5.00		212.43 ± 7.83	**173.19 ± 6.28**	**	**196.85 ± 4.03**
Max AP rising slope (V/s)[4]	**814.80 ± 20.79**	*	**727.94 ± 27.35**	**974.25 ± 32.66**	**	**768.69 ± 16.85**
Max AP decay slope (V/s)[5]	517.11 ± 17.51		503.61 ± 26.90	**677.31 ± 25.19**	**	**558.26 ± 17.75**

AP properties are represented as mean ± SEM of medians of parameters measured from the first AP of the first current-induced AP train above 250 Hz. AP, action potential.

[1]Membrane potential at the point where AP rate of rise surpassed 50 V/s
[2]Voltage difference between AP threshold and peak
[3]Full width at half maximum amplitude
[4]Maximum rate of rise during the AP rising phase
[5]Maximum rate of decay during the AP decaying phase

* = $p<0.05$; ** = $p<0.01$
